# Effectiveness of Social Media-Based Interventions for the Promotion of Physical Activity: Scoping Review

**DOI:** 10.3390/ijerph182413018

**Published:** 2021-12-10

**Authors:** Liane Günther, Sarah Schleberger, Claudia R. Pischke

**Affiliations:** Institute of Medical Sociology, Centre for Health and Society, Medical Faculty, University of Düsseldorf, Moorenstraße 5, 40225 Düsseldorf, Germany; Sarah.Schleberger@hhu.de (S.S.); claudiaruth.pischke@med.uni-duesseldorf.de (C.R.P.)

**Keywords:** social media, intervention, physical activity, scoping review, behavior change

## Abstract

A global target of the World Health Organization (WHO) is to reduce physical inactivity among all adults and adolescents by approximately fifteen percent by 2030. Social media could have an impact in this effort because of its enormous reach, potentially addressing underserved populations in need for physical activity (PA) interventions. This scoping review provides a broad overview of social media-based interventions and systematically maps the evidence regarding their effectiveness for PA promotion and other health outcomes. Scopus and Medline were searched using the terms “physical activity” and “social media” and the names of key social media platforms. Following the PRISMA guidelines for scoping reviews, abstracts and full texts were screened for eligibility. In total, 12,321 publications were identified and 53 met the inclusion criteria. The use of Facebook was most prevalent in PA interventions, followed by study-specific platforms. More than one third of the studies revealed positive effects regarding the promotion of PA. Additionally, social media-based interventions positively affected other physical dimensions of health (e.g., weight or blood pressure). Results pertaining to feasibility were heterogeneous. Social media seems to be a promising tool for increasing PA at the population level. Future studies should take the abundance of platforms into account and select social media platforms consciously.

## 1. Introduction

### 1.1. Background

Physical inactivity is a leading risk factor for mortality worldwide and contributes to the etiology and progression of non-communicable diseases (NCDs), such as cardiovascular disease, type 2 diabetes, various types of cancer, and chronic respiratory diseases. Seventy-one percent of all global deaths in 2016 were attributed to NCDs [1,2]. In order to prevent and minimize the burden of NCDs, the World Health Organization (WHO) developed the Global Action Plan 2013–2020 with nine global targets to be accomplished by the year 2025. One of the targets is to reduce physical inactivity worldwide by ten percent [3].

A status update of the WHO from 2020 suggests that, while some progress has been made to reduce the burden of NCDs (e.g., to decrease NCD-related premature deaths), further effort is required to reduce physical inactivity because of an insufficient success in equally addressing all NCD risk factors, as indicated by a rising prevalence of obesity [2]. Particularly in low- and middle-income countries, challenging barriers to the implementation of effective interventions are frequently encountered. However, even in economically developed countries, levels of physical inactivity have increased in the past decades as a result of urbanization, digitalization, and varying options of transportation. According to the 2030 Sustainable Development Goals set by the United Nations in 2015, the WHO refined the Global Action Plan and incorporated a system-based approach to ensure that all people, no matter which age, gender, socioeconomic status, disability status, or geographical origin, have accessible opportunities for being physically active in their daily lives. The new global target is to reduce physical inactivity in all adults and adolescents by approximately fifteen percent by the year 2030 [4].

Social media holds great potential for the accessible promotion of physical activity (PA). Based on the synthesis of definitions of social media appearing from 2007 onwards, Mróz-Gorgoń and colleagues (2016) defined social media as “network community communication channels” (p. 38), which allow the exchange of information, interaction and integration on a private and commercial domain [5]. Due to the rapid development of social media, a multitude nowadays exists, for example, discussion forums, wikis, audio platforms (e.g., Clubhouse, Spotify), live streams (e.g., Twitch), blogs (e.g.,Tumblr), social networking sites (e.g., Facebook), and media sharing networks (e.g., Instagram, YouTube) which are not listed under the 2016 definition. All of these platforms have participatory features in common that facilitate the exchange with existing friends, like-minded people, communities with similar characteristics (e.g., patient communities), or brands. Results of several systematic reviews revealed that this interpersonal engagement with other users fostered via typical features of social media, such as bulletin boards and chats, effectively promotes knowledge regarding health topics [6]. Another advantage is that via these built-in social elements, supportive, encouraging, or even competitive interactions can take place easily. Both social support and social comparison can motivate individuals to engage in more PA. A study by Cavallo and colleagues, 2014, found that social support via Facebook stemming from existing friendships was more likely to change PA behavior than communication about PA in a dedicated Facebook group [7]. Results from Zhang et al., 2016, indicated that adding a competitive element to these supportive networks could have an even more pronounced positive effect on exercise levels [8]. 

Beyond that, social media has an enormous reach; it is estimated that the number of active social media users worldwide reached more than 3.6 billion users in 2020, and this trend is continuing to rise [9]. Due to the global technological progress and gains in internet inclusion in nine of the fifteen low-income countries, the access is no longer reserved for privileged people from high-income countries [10]. Taking into account recent user data, social media appears to have the potential to address underserved populations known to have restricted access to health care, due to physical, geographical, financial, or educational barriers [11]. Instagram mainly attracts young people, and Facebook and YouTube reach between 64 and 70% of people with lower levels of education. Additionally these two platforms have, with nearly 50%, the most users who are above the age of 65 years, as well as a large proportion of residents from rural areas (i.e., 67–74%) [12].

Although previous evidence indicates that social media-based interventions for the promotion of PA achieve high levels of engagement and retention [13], results regarding their overall effectiveness for increasing PA remain inconclusive. In the WALK 2.0 trial, Kolt and colleagues, 2017, detected a short-term increase in moderate to vigorous physical activity (MVPA) in an intervention group offered PA promotion via an interactive Web 2.0 site compared to a less interactive Web 1.0 group [14]. In contrast, Edney and colleagues, 2020, found no significant effect of their gamified, social networking intervention ‘Active Team’ on MVPA [15]. The application of popular online-social networks, such as Facebook, also yielded contradictory results in past studies. While one study did not find any significant changes in PA in a group including female undergraduate students that used Facebook compared to a control group receiving general educational material after twelve weeks [16], another study observed a significant increase in steps walked per day in a Facebook social support group compared to a standard walking group after eight weeks [17]. Considering that social media is quickly evolving and may hold potential for PA promotion, the overall aim of this scoping review is to provide a broad overview of existing social media-based interventions and map the evidence regarding their effectiveness for the field of PA promotion.

### 1.2. Objectives

This scoping review was conducted in order to:

1. Identify which social media platforms are used in interventions for the promotion of PA to date.
2. Systematically map the evidence regarding their effectiveness for PA promotion.
3. Determine which dimensions of health are captured as secondary outcomes in the studies included in the scoping review.
4. Summarize the evidence regarding the impact of social media-based PA interventions on these secondary health outcomes.
5. Summarize the evidence regarding acceptability, use, and usability of social media-based PA interventions.

## 2. Methods

### 2.1. Protocol and Registration

According to the PRISMA guidelines for scoping reviews (PRISMA-ScR) [18], a protocol was developed a priori and is accessible on Open Science Framework [19].

### 2.2. Eligibility Criteria

Due to the relatively broad nature of a scoping review and the explorative review questions, all published literature on social media based-interventions promoting PA was included. 

Social media based-interventions encompass all interventions delivered via any type of social media or at least incorporate one social media component of either existing, popular platforms or stand-alone platforms (e.g., Facebook group, discussion forums, message boards) to promote PA.

All studies that compared the social media-based condition to either (A) a non-social media-based intervention (e.g., a face-to-face intervention, programs delivered via websites or mobile apps, mass media campaigns, video tutorials), (B) a control group not exposed to any intervention, or (C) a no-comparator condition were included. Additionally, articles needed to address PA (or conversely physical inactivity, including sedentary behavior) as a primary outcome to be included, regardless of whether PA was assessed using objective or subjective methods of measurement. PA outcomes were considered as measures of PA volume reported in various units (e.g., steps, min/day, number of repetitions).

All types of studies carried out on the research topic, so far, were eligible for inclusion (e.g., experimental and quasi-experimental designs, pilot and observational studies, reviews and meta-analysis). Furthermore, inclusion was restricted neither to any specific population, as long as study participants were human beings, nor to contexts in which studies were conducted. Conference proceedings, abstracts without full texts, and study protocols without results were excluded from this review. In addition, systematic reviews that did not provide complementary information, because they contained only a few studies on social media-based interventions, which were already included in the data extraction for this scoping review, were excluded.

### 2.3. Information Sources

To identify relevant literature, the electronic databases Scopus and Medline via PubMed were searched on 4 May and 12 May 2020 by the two authors LG and SS. The search was comprehensive and limited by neither publication date nor publication type or any other filters. Reference lists of the included full texts were scanned for potentially relevant other articles.

### 2.4. Search

The search strategy was peer-reviewed and refined by an information scientist from the Cochrane Metabolic and Endocrine Disorders Group before implementation. We searched the concepts “physical activity” and “social media” as MeSH terms or index terms and key words, as well as their appropriate synonyms. In addition, we searched key social media platforms from 2014–2020 by name [9]. The search terms were combined by using the Boolean operators AND and OR. Detailed information on the search strategy for both databases is provided in Supplementary 1.

### 2.5. Selection of Sources of Evidence

The selection of sources of evidence was executed in two steps after all identified records had been exported to EndNote X9.1 and Rayyan. First, two authors (LG, SS) screened titles and abstracts of all identified records independently, using the web app Rayyan. Disagreements concerning inclusion/exclusion for full text screening were discussed with a third author (CRP) until they were resolved. Second, full texts of all records that were deemed relevant after screening phase one, were obtained. Whenever a full text was not accessible, the corresponding author was contacted for retrieval. Then, the two authors LG and SS independently screened all full texts for eligibility. Any discrepancies were resolved via discussion with the third reviewer (CRP).

### 2.6. Data Charting Process

For the extraction of all relevant information from the included sources, a data charting sheet was developed similar to the one provided by Elm and colleagues, 2019 [20], and pilot-tested in advance, by two authors (LG, SS). Each of them extracted data from five randomly chosen articles of the pool of included articles. In a discussion, the process of data charting with this abstraction tool was evaluated and the tool was adapted, where necessary. Then, the two authors LG and SS independently charted data from each eligible full-text by using the revised data charting sheet, formatted in Microsoft Word (Supplementary 2). After completion of the data charting process, ten articles were randomly selected and the two reviewers compared their corresponding data charts and verified the accuracy of their extraction based on the a priori protocol and the original full text. In cases of differences in the extracted data between the two authors, all data charted were compared again, and the third author (CRP) was consulted to resolve inconsistencies.

### 2.7. Data Items

Data on the general information of the reference (e.g., title, authors, country, publication type), evidence source characteristics (e.g., participants, sample size, withdrawals), the intervention (e.g., clusters, content, duration), outcomes (type, definition, unit, measurement tools), and results (e.g., comparison, statistical results) were captured. Additional information, such as the key conclusion of the study, notes or correspondence with the lead or senior authors, was provided at the end of the charting form.

### 2.8. Synthesis of Results

First, the included references were grouped by study type (randomized controlled trials, pilot studies, quasi-experimental and observational studies, and systematic reviews). Additionally, articles were grouped by type of social media to obtain an overview of studies using social media platforms as stand-alone interventions, specifically designed for the purpose of the study, versus those which incorporated well-known social media applications into interventions but also included other components. Along with the main objective of this scoping review, data on how PA was quantified were charted, and studies were summarized according to their effectiveness for improving PA (dependent on reported *p* values for in-between group differences or withingroup differences from baseline to follow-up). Secondary health outcomes were grouped into various dimensions of health examined and by effectiveness. Additionally, the evidence on feasibility was summarized, (e.g., descriptive results on use, acceptability, and usability of interventions).

## 3. Results

### 3.1. Selection of Sources of Evidence

**Figure 1:**
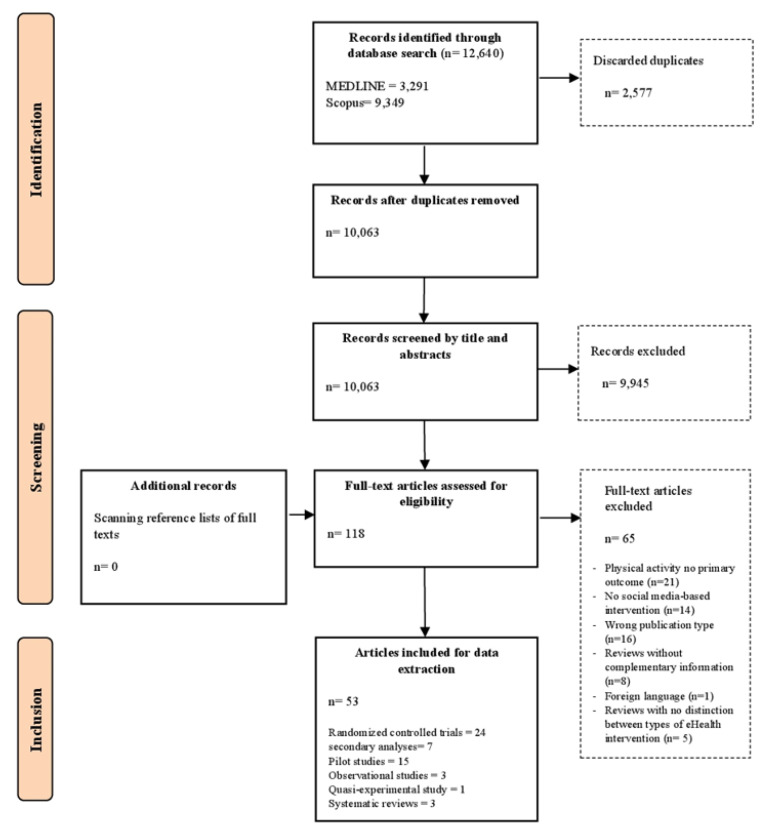
Flow chart of the selection process.

The initial database search generated 12,321 hits. Another 319 records were found via search alerts in the two databases before the screening process ended and were added to the final results. A total of 2064 duplicates were detected by Endnote and deleted before uploading the remaining records in Rayyan. Via a manual search, another 513 duplicates were identified by the two authors LG and SS during the screening process and subsequently deleted. This resulted in 10,063 total records, which were screened by title and abstract. After the first screening phase, 9945 records were excluded, and the full texts of the remaining 118 publications were screened for eligibility. Throughout this second screening phase, another 65 articles were excluded, with the most common reasons for exclusion being: PA was not a primary outcome (*n* = 21), no social-media based intervention was examined (*n* = 14), and the publication type was wrong (e.g., study protocols, conference proceedings, *n* = 16). After completion of the screening process, 53 articles referring to 43 studies/trials were included in this scoping review. For detailed information about the selection process, see Figure 1 (flow chart).

### 3.2. Characteristics of Sources of Evidence

The search delivered 24 randomized controlled trials (RCTs) [8,14,15,16,17,21,22,23,24,25,26,27,28,29,30,31,32,33,34,35,36,37,38,39], 15 pilot studies [40,41,42,43,44,45,46,47,48,49,50,51,52,53,54], 3 observational studies [55,56,57], and 1 quasi-experimental study that compared two groups but did not randomize its participants [58], and 3 systematic reviews [13,59,60]. The seven remaining publications presented results of secondary data analyses based on the included RCTs [61,62,63,64,65,66,67]. Detailed information on the study characteristics is presented in Supplementary 3.

Overall, the 43 studies included 11,469 participants, ranging from a minimum of 10 to a maximum of 5010 individuals [48,56]. Participants were between the ages of 11 and 65 years [29,37], predominantly Caucasian (except for Joseph et al., 2015, including only African American women [25]), and either predominantly or exclusively female, except for two studies that only included men [27,47].

Maher et al., 2014, included a total sample of 113,988 participants across ten studies in their systematic review [59]. The other two reviews provided data on neither sample size nor sample characteristics [13,60].

In 28 studies, a pre-existing social-media platform was used for intervention delivery [16,17,21,25,28,30,31,32,33,34,35,36,38,40,41,43,45,46,48,49,50,51,53,54,55,56,57,58], whereas in eleven studies, a study-specific platform was developed exclusively for the purpose of the study [8,14,22,23,24,27,37,39,42,47,52]. Lastly, four studies incorporated a combination of a specifically designed app with the well-known social-media platform Facebook [15,26,29,44]. Forty studies implemented a social networking site or social networking feature, with Facebook being the most commonly used by itself (*n* = 18) [16,17,21,25,28,31,33,35,36,38,43,45,48,49,50,51,53,54]. Other well-known social networking platforms were the Ning network (*n* = 1) in combination with Facebook [32], Meetup (*n* = 1) [34], and the Wanbu network (*n* = 1) [56]. The social networking feature of the Fitbit App was utilized in two studies [30,40]. Of the eleven studies that solely used their tool developed for the study, five had designed an app [24,27,37,47,52] and four had implemented a website, all of them including social networking features [8,14,22,39]. Two studies used social networking features, which remained unspecified in the respective articles [23,42]. The microblogging platform Twitter was used in two studies [41,46], one of them combined it with Facebook [41]. Another study used YouTube in combination with the online social network Instagram [58], and two investigated the virtual game Pokémon Go [55,57]. In the three systematic reviews, a wide variety of social media platforms was applied across the several studies, whereas Petersen et al., 2019 [13], summarized studies that incorporated Facebook only, Maher et al., 2014, and Williams et al., 2014, included trials examining the role of interventions using the popular platforms Facebook and Twitter, as well as study-specific online health social networks or discussion boards [59,60].

The RCTs conducted were either two-armed (*n* = 14) [16,17,21,22,23,25,27,28,29,30,31,34,35,36], three-armed (*n* = 7) [14,15,26,32,33,37,39] or even four-armed (*n* = 3) [8,24,38] interventional studies with comparator conditions or a waitlisted control group. The pilot studies either analyzed a single group (*n* = 8) [40,41,43,44,48,51,52,53] or had a two-arm study-design (*n* = 7) [42,45,46,47,49,50,54]. Intervention content varied substantially among the included RCTs and pilot studies. Most often, social media was used as a tool for the distribution of information and recommendations regarding PA. Secondly, social media enabled the sharing of PA data on an individual or team basis and reinforced interactions between intervention/study participants or with researchers via discussion boards or groups. Most studies argued that the interaction feature was a means of either providing a source of social support, motivation, comparison or accountability for PA, or a combination of all of these features. Other common features were goal setting and feedback on individual or team progress. The huge majority of the studies combined the use of this digital component with a measurement device (e.g., accelerometer, pedometer) to track PA and other intervention add-ons, such as e-mails, in-person meetings, or exercise classes. In the control groups of the studies, participants usually received comparator interventions, such as educational classes, an almost identical intervention without the social media component, or they were waitlisted.

Two observational studies tracked data of users and non-users of Pokémon Go [55,57], an augmented reality game that rewards PA in a gamified context, and one study analyzed the use of the Wanbu network, a platform onto which step data can be uploaded and discussed with friends [56].

In the one and only quasi-experimental study, a YouTube exercise video was delivered to both the intervention and control group. The intervention group obtained additionally motivation via Instagram [58].

Of the three systematic reviews, two summarized evidence on the effectiveness of interventions delivered via a social media platform for changing PA and other health outcomes, such as diet and weight [59,60], and quality of life [59]. The third review focused on PA and psychosocial constructs related to PA and compared the effectiveness of mobile apps with and without social networking features [13].

In all of the included articles, PA was the primary outcome, but it was captured in numerous ways. Most of the studies (*n* = 23) reported the minutes of PA executed at a certain level of intensity, better known as time spent with moderate to vigorous PA (MVPA) or sedentary time; time spent on either light, moderate, or vigorous PA; and total weekly PA [14,15,23,25,26,27,28,29,31,32,33,36,40,44,45,46,47,48,49,50,53,54,55]. Further, eighteen studies quantified PA in steps per hour, day, or week [14,17,21,25,31,34,37,40,42,44,46,47,48,51,52,53,56,57]. Another five studies reported energy expenditure and metabolic equivalents (METs) [30,31,35,38,48]. Less frequently used measures of PA were active travel (*n* = 1) [41], number of attended exercise classes (*n* = 3) [8,39,58], physical fitness (*n* = 1) [43], and exercise frequency (*n* = 2) [16,22]. Some studies captured the time spent performing a specific type of PA, such as walking (*n* = 5) [23,26,29,44,55], or the time spent in plank position (*n* = 1) [24]. In most studies, at least two measurements were performed to quantify PA.

Across the three systematic reviews, only Peterson et al., 2019, reported the PA outcomes of interest in this scoping review in detail (e.g., light, moderate, vigorous PA, daily steps, and sedentary behavior) [13], whereas Maher et al., 2014, and Williams et al., 2014, referred to PA in general [59,60].

Secondary health outcomes were measured in 30 studies investigating between one and eleven outcomes. The majority of studies captured aspects of physical health via anthropometric measures, such as height and weight in order to determine body-mass index (*n* = 10) [14,21,22,25,26,32,33,47,53,55] or only weight (*n* = 11) [17,21,23,27,31,36,47,48,49,50,53]. Others assessed fat mass or the percentage of body fat (*n* = 8) [21,22,27,31,46,48,49,50], hip/waist circumference (*n* = 6) [17,21,27,32,47,53], blood pressure (*n* = 4) [21,46,47,53], and serum lipids (e.g., total cholesterol and triglycerides, *n* = 2) [21,23]. Physical fitness was also a commonly included outcome with five studies measuring cardiorespiratory fitness [28,31,48,49,50], two studies investigating aerobic fitness [27,32] and one focusing on grip strength [27]. Another three studies captured health behaviors, such as eating habits, nutrition, and alcohol consumption [47,49,53]. Psychosocial outcomes related to PA that were tracked in the included studies were motivation, enjoyment, perceived barriers, social support, self-efficacy, group cohesion and outcome expectancies or combinations of these outcomes were reported in fourteen studies [24,25,28,31,34,36,37,38,45,48,49,50,53,58]. Eleven studies measured mental health outcomes, such as quality of life, mood, depressive symptoms or signs of anxiety or stress, psychological well-being, vitality, and sleep [14,15,26,29,31,36,38,45,47,48,51]. Singular and more specific outcome measures were dog’s PA (*n* = 1) [34], attention deficit hyperactivity disorder (ADHD) symptoms (*n* = 1) [51], and premenstrual syndrome (PMS) symptoms (*n* = 1) [30].

Overall, 29 studies examined the use, acceptability, and usability of the interventions [15,16,17,24,25,26,27,28,29,31,32,34,36,37,40,41,42,43,44,45,48,49,50,52,53,54,56,58,61]. Twenty-four studies reported results on use pertaining to the social media components (e.g., number of posts, number of logins, step entries) [16,17,25,26,27,28,29,31,32,34,36,37,40,41,42,44,45,48,50,52,53,54,56,61]. Acceptability was assessed either regarding the entire program (*n* = 11) [24,25,26,32,34,36,40,43,53,54,58] or the digital component only (*n* = 10) [25,27,28,29,31,36,48,49,50,53]. Usability (*n* = 8) was commonly determined via rating scales or evaluation forms mainly focused on tracking how easy the intervention (component) was to use [15,31,42,44,49,50,52,61]. In two of the included systematic reviews, results on use and engagement with the intervention materials across the included studies were synthesized [13,59].

### 3.3. Results of Individual Sources of Evidence

Individual results on PA and the dimensions of health captured as secondary outcomes in the included studies are displayed in Supplementary 3.

### 3.4. Synthesis of Results

In total, 24 studies demonstrated positive effects concerning PA [8,15,17,21,23,25,26,28,29,30,35,36,39,41,43,44,46,47,51,53,55,56,57,58]. Sixteen studies found significant between-group differences in favor of the intervention group that was exposed to a social media condition [8,15,17,21,23,25,28,29,30,35,36,39,46,47,55,58]. Another eight studies reported significant improvements in PA over time within groups (from baseline to follow-up) [26,41,43,44,51,53,56,57]. 

Conversely, in the WALK 2.0 study [67], PA increased by 92.8 min/ week more in the intervention group that used standard Web 1.0 features compared to the group that used Web 2.0 features. At the three-month follow-up, 77% of the Web 1.0 participants were sufficiently active compared to 71.5% of the Web 2.0 participants. In the study conducted by van Woudenberg et al., 2020, the waitlisted control group showed even greater improvement in total PA than the group which was exposed to vlogs [37].

Considering the evidence reported in the three systematic reviews included in this scoping review, Petersen et al., 2019, showed in their systematic review that combined social networking apps were less effective in increasing PA (*n* = 3) than conventional standalone apps (*n* = 7) [13]. In the review by Williams et al., 2014 [60], only one of five studies found that PA was positively affected by a social media-based intervention, whereas Maher et al., 2014, found in their review that of four studies, three had a sufficient, two a moderate, and one a high effect size [59].

Physical aspects of health, such as weight [23], BMI [47,67], body fat [46], waist circumference, eating behavior [46,47], blood pressure [47], and triglycerides [47] were improved effectively in favor of the intervention groups employing social media in four studies. Another two studies reported improvements within groups from pre- to post-measurement in waist circumference [17], as well as fruit and vegetable intake [53]. Chee et al., 2014, provided evidence that increasing PA was beneficial for improving components of the metabolic syndrome (e.g., HDL cholesterol, blood pressure, triglycerides, waist circumference, fasting glucose) [21]. Regarding psychosocial aspects of health, the results were contradictory. Five studies found that enjoyment [38], group cohesion [24], motivation [45], social norms [37], self-regulation, and social support from family members [25] were positively affected in the groups that participated in a social media based- intervention compared to controls. In contrast, two studies found that social support and self-efficacy were lower among participants of the intervention groups and perceived barriers to higher PA [31,66]. Schoenfelder et al., 2017, reported improvements in ADHD symptoms from baseline to 4-week follow-up of a Fitbit intervention supported by a Facebook group [51].

Only one study found significant effects of an exercise program that was supplemented by a social network on health-related quality of life [47]. A second study found that individuals who were more physically active scored higher on the scale for health-related quality of life [64]. Furthermore, Nam et al., 2020, provided evidence that a range of PMS symptoms (e.g., fatigue, anxiety) that contribute to all dimensions of health decreased in a social media-based support group compared to a control group [30].

Summarized evidence on secondary health outcomes analyzed in the three included systematic reviews was marginal. Whereas Williams et al., 2014, found that dietary fat consumption decreased significantly in social media-supported study conditions [60], Maher et al., 2014, found an effect on weight change across three studies with a varying effect size from negligible to large [59]. In the third review, only one included study revealed improvements in the assessed psychosocial outcomes (e.g., self-efficacy and exercise motivation) over time [13].

Two-third of the included studies and two of the systematic reviews reported results pertaining to feasibility. Due to the great heterogeneity in the assessment of acceptability, use, and usability across the different references, pooling of the data was difficult. Nevertheless, it was noticeable that across six studies the use of Facebook as a motivator for PA was rated positively by study participants [25,28,31,49,50,53,62], whereas the usability of three study-specific networks was rated poorly [15,27,52]. 

Descriptively reported results regarding the primary or secondary outcomes are shown in Supplementary 3.

## 4. Discussion

### 4.1. Summary of Evidence

This scoping review identified 53 publications with findings from 43 studies and three systematic reviews on the promotion of PA via social media-based interventions. Social networking sites were used most frequently for the promotion of PA. In the twenty-eight studies and the three systematic reviews evaluating the role of pre-existing social networking sites, the use of Facebook was most prevalent. Secondly, the incorporation of a developed standalone, health-focused online social network was very common. Four studies combined the use of a study-specific social networking site with Facebook. Regarding the other types of social media, only pre-existing applications were utilized, in particular, Twitter, Pokémon Go, and YouTube. 

Most studies included in this scoping review were RCTs followed by pilot studies, of which 34 were at least two-armed and compared their intervention content ranging from simple recommendations for PA to fostering social interactions facilitating PA or making PA enjoyable and measurable, to another condition or a waitlisted control group. Comparator conditions usually consisted of identical intervention content without the social media (social interaction) component. Additionally, three systematic reviews were included in this scoping review. In sum, more than three quarter of the studies employed study designs allowing for an estimation of intervention effects.

More than one third of the studies revealed positive effects on PA. Most frequently, PA was improved due to a social media-based intervention when compared to an alternative intervention in eleven cases, and five studies showed increases in PA in comparison to a waitlisted control group. Evidence from the systematic review was, in part, inconclusive. Here, only seven studies were identified in which PA was effectively increased through social media, whereas seven studies found effects in favor of conventional apps.

Nearly two-thirds of the articles included covered other dimensions of health as secondary outcomes. These addressed, in addition to the physical dimension, psychosocial and mental aspects of health. Here, social media-based interventions had the most positive impact on physical parameters of health related to weight/body composition or cardiovascular parameters, as they were predominantly captured. Psychosocial parameters of health were less frequently measured, and results were contradictory. Only a handful of studies could demonstrate that social media-based interventions had beneficial effects on PA by fostering motivation, social support, and self-efficacy. A positive effect on psychological and health-related quality of life, the most recorded mental health outcome, was evident in only one study that incorporated social media.

A range of results concerning acceptability, use, and usability of social media-based PA interventions were uncovered in this scoping review and it is impossible to draw any generic conclusion regarding the feasibility of such interventions. The results should be interpreted study by study and with caution taking their different ways of assessing feasibility into account.

One of the main findings, of this work is that the most commonly incorporated platform in PA interventions is Facebook. Given the rapid development of a variety of social media platforms during the last decade, it can be argued that a broad spectrum of innovative social media have not yet been incorporated and scientifically tested. Future intervention studies should take advantage of this abundance of platforms and select them consciously, according to the requirements, needs, and preferences of the individual target populations. This is supported by a scoping review of Lee and colleagues, 2019, concluding that mobile apps that focus on health behavior change have to be appropriate for their target sample [68]. In line with this argument, a specifically modified app and social network for overweight hockey fans demonstrated a high retention and significant improvements in PA when compared to a waitlisted control group [47]. 

It is important to emphasize that there is no fundamental need for the development of new platforms, but relying on pre-existing applications and using them purposefully will be sufficient for future health behavior change interventions. Emerging evidence provides some indication that social media can be effective for improving PA and other physical parameters of health. However, there is limited research about the efficacy of social media concerning other secondary health outcomes necessitating additional research.

Despite those positive findings, the utilization of social media in health care and prevention should be very carefully considered and always critically questioned. Although the availability of the internet has increased widely worldwide, mostly via technological improvements (e.g., broadband quality) [10], social inequalities continue to exist. The so-called digital divide has shifted from a lack of material resources over a shortcoming of skills to use them to an absence of personal capacities transferring consumed information of the World Wide Web into favorable behavior, better known as third-level digital divide [69]. Recent study findings from Finland suggest that especially disadvantaged populations which should be the target of eHealth intervention approaches and services, such as older people and those with a low socioeconomic status, poor health, or socially isolated persons are at the highest risk for digital exclusion because ICT-based health care seems less beneficial for them [70]. Furthermore, the use of social media can affect health negatively, as was lately shown in a cross-sectional survey analysis by Henzel et al. 2021, [71], who found out that addictive social media use is prevalent in the young generation and is related to mental distress and other addictive behaviors such as gaming. Another harmful effect of social media that has become a challenging public health issue is the occurrence of fake news that causes fear, uncertainty, and social division, as seen in the current COVID-19 pandemic. Misleading information spread via social media has the potential to influence health and wellbeing negatively at a global level [72].

### 4.2. Limitations

A key limitation of the scoping review is the heterogeneity of the included literature concerning methodology and outcome measures used in the studies. This resulted in difficulties in pooling quantitative data and evaluating them appropriately as envisaged to understand the effects of social media-based interventions on PA and health, and, in particular, interpreting their feasibility. Furthermore, it could be argued that restricting the search to two literature databases is another limitation, because relevant studies may have been missed. Because a scoping review does not assess the risk of bias, conclusions about the quality of the included sources of evidence cannot be drawn, and the generalizability of the described findings remains unclear. Another limitation is that the included studies were conducted prior to the emergence of the COVID-19 pandemic. The role of social media for PA promotion may have somewhat changed under pandemic conditions, possibly even becoming increasingly important, as gyms and sports facilities were temporarily closed during lockdowns. However, this aspect will have to be analyzed in future scoping reviews, including studies conducted and published after the spring of 2020.

## 5. Conclusions

To our knowledge, this is the first scoping review which provides a broad overview of the incorporation of the latest social media technologies in research on PA promotion. Taking into account key social media platforms from 2014–2020, this work is up-to-date concerning recent Web 2.0 approaches to prevent and minimize the burden of physical inactivity and NCDs, but continuous updates will be essential considering the fact that social media is a quickly evolving field. Future effort in this line of research is required to attain an increased standardization of these indicators and measures and an overarching public health framework that can be used to appraise the feasibility of future social media interventions in health promotion and care.

## Data Availability

The data are contained as Supplementary Materials, see Supplementary 1–3.

## Supplementary Materials

The search strategy, the data charting sheet, and information on the study characteristics are available online at https://www.mdpi.com/article/10.3390/ijerph182413018/s1, Supplementary 1: Search strategy, Supplementary 2: Data charting sheet, Supplementary 3: Study characteristics.

## Abbreviations

ADHD	Attention Deficit Hyperactivity Disorder
BMI	body mass index
MeSH	medical subject headings
METs	metabolic equivalents
MVPA	moderate to vigorous physical activity
NCDs	non-communicable diseases
PA	physical activity
PMS	premenstrual syndrome
RCTs	randomized controlled trials
WHO	World Health Organization
