# Supplementary material for: Effectiveness of Social Media-Based Interventions for the Promotion of Physical Activity: Scoping Review
Source: Int J Environ Res Public Health. 2021 Dec 10;18(24):13018. doi: 10.3390/ijerph182413018 (PMC8702047; doi:10.3390/ijerph182413018)
Supplement: Supplementary file 1 [file ijerph-18-13018-s001.zip › ijerph-1469611-supplementary.pdf]

Supplementary 1: Search strategy

1. Search terms

physical activity:

- physical activ*
- motor activ*
- exercise*
- sport*
- types
  (walk*, jogg* swim*, weight lift*, danc*, aerobic*)
  (circuit or weight or aerobic or cross or endurance or resistance train*)

social media:

- social media
- social network*
- online social network*
  online social platform*
- Web 2.0
- names
  (Facebook, Instagram, Twitter, YouTube, TikTok, Qzone, Tumblr, Google+, LinkedIn, YY, reddit, VKontakte, Pinterest, Sina Weibo, Baidu Tieba, Kuaishou)

key social media platforms:

- Facebook
- Instagram
- Twitter
- YouTube
- TikTok
- Qzone
- Tumblr
- Google+
- LinkedIn
- YY
- reddit
- VKontakte
- Pinterest
- Sina Weibo
- Baidu Tieba
- Kuaishou

* according to "Key global social platforms, by active users worldwide 2014-2020"; Hootsuite & We Are Social (2014-2020), "Digital 2014-2020: Global Digital Overview," retrieved from https://datareportal.com/

2. Medline search (via Pubmed on 4th May 2020)

Search name	Search query	Type of search	Results
#1	(physical activ*[tiab] OR motor activ*[MeSH Terms] OR motor activ*[tiab] OR exercise*[MeSH Terms] OR exercise*[tiab] OR "physical education and training"[MeSH Terms] OR ("physical education"[tiab] AND training[tiab]) OR sport*[MeSH Terms] OR sport*[tiab] OR physical fitness[MeSH Terms] OR walk*[tiab] OR jogg*[tiab] OR swim*[tiab] OR “weight lift*”[tiab] OR danc*[tiab] OR aerobic*[tiab] OR "circuit train*"[tiab] OR "weight train*"[tiab] OR "cross train*"[tiab] OR "endurance train*"[tiab] OR "resistance train*"[tiab])	MeSH terms + Title Abstract	838,868
#2	("social media"[MeSH Terms] OR "social media"[tiab] OR "social network*"[Mesh] OR “social network*”[tiab] OR “online social platform*”[tiab] OR “Web 2.0”[tiab] OR Facebook[tiab] OR Instagram[tiab] OR Twitter[tiab] OR YouTube[tiab] OR TikTok[tiab] OR Qzone[tiab] OR Tumblr[tiab] OR Google+[tiab] OR LinkedIn[tiab] OR YY[tiab] OR reddit[tiab] OR VKontakte[tiab] OR Pinterest[tiab] OR “Sina Weibo”[tiab] OR Baidu Tieba[tiab] OR Kuaishou[tiab])	MeSH terms + Title Abstract	56,230

Search name	Search query	Results
#3	#1 AND #2	3,201
notes:<br>Kuaishou not found		

3. Scopus search (on 12th May 2020)

Search name	Search query	Type of search	Results
#4	TITLE-ABS(physical activ*) OR TITLE-ABS(motor activ*) OR TITLE-ABS(exercise) OR TITLE-ABS("physical education" AND training) OR TITLE-ABS(sport) OR TITLE-ABS(walk*) OR TITLE-ABS(jogg*) OR TITLE-ABS(swim*) OR TITLE-ABS("weight lift*") OR TITLE-ABS(danc*) OR TITLE-ABS(aerobic*) OR TITLE-ABS("circuit train*") OR TITLE-ABS("weight train*") OR TITLE-ABS("cross train*") OR TITLE-ABS("endurance train*") OR TITLE-ABS("resistance train*")	Title Abstract	1,481,653
#5	TITLE-ABS("social media") OR TITLE-ABS("social network*") OR TITLE-ABS("online social platform") OR TITLE-ABS("Web 2.0") OR TITLE-ABS(Facebook) OR TITLE-ABS(Instagram) OR TITLE-ABS(Twitter) OR TITLE-ABS(YouTube) OR TITLE-ABS(TikTok) OR TITLE-ABS(Qzone) OR TITLE-ABS(Tumblr) OR TITLE-ABS(Google+) OR TITLE-ABS(LinkedIn) OR TITLE-ABS(YY) OR TITLE-ABS(reddit) OR TITLE-ABS(VKontakte) OR TITLE-ABS(Pinterest) OR TITLE-ABS("Sina Weibo") OR TITLE-ABS(Baidu Tieba) OR TITLE-ABS(Kuaishou)	Title Abstract	245,788

Search name	Search query	Results
#6	#4 AND #5	9,120

4. Medline & Scopus alerts (on 7th June 2020)

Search name	Search query	Results
#7	#3 AND #6	319 (#3= 90; #6= 229)

5. Total

#8	#3 + #6 + #7	12,640

Supplementary 2

Study selection & Data extraction form (trials & reviews)

**Evidence source information**	
**Date form completed**	
**Name/ID of person extracting data**	
**Authors**	
**Title**	
**Citation details** (e.g. year, journal, volume, issue, pages, DOI	
**Country**	
**Publication type**	
**Study funding sources**	
**Possible conflicts of interest**	
**Notes**	

**Study Eligibility**	
**Aims/Objectives**	
**Intervention/s**	
**Outcome measures**	
☐ **INCLUDE**<br><br>☐ **EXCLUDE**	
**Reason for exclusion**	

DO NOT PROCEED IF REFERENCE EXCLUDED FROM REVIEW

Evidence source characteristics	
**Participants** (e.g. age, sex, other socio-demographic characteristics, health status & co-morbidities)	
**Total no. recruited**	
**Withdrawals**	
**Exclusions**	
**Included in final analysis**	
**No. of missing participants and reasons**	
**Design**	
**Notes**	
**Intervention/s**	
**Cluster/s**	
**No. randomised to each cluster**	
**Description**	
**Duration of intervention/s**	
**Timing**	
**Delivery**	
**Providers**	
**Notes**	

**Outcomes**	
**Outcome name, definition & unit**	
**Explanatory outcomes**	
**Time points measured**	
**Time period measured**	
**Time points reported**	
**Measurement tool**	
**Imputation of missing data**	
**Assumed risk estimate/risk of bias score**	
**Power**	
**Notes**	
**Results**	
**Comparison** (interaction effects)	
**Results**	
**Any other results reported**	
**Statistical methods used**	
**Notes**	
**Other information**	
**Key conclusion of authors**	

**Correspondence required for further study information**	
**Does the study/review directly address the scoping review objectives?**	
**Notes**	

Supplementary 3

1

2	a. Author(s)	b. Year of publication	c. Study location	d.Title	e. Aims/purpose	f. Study population (age, gender, race, disease)	g. Number of participants (n)
3	a) Alley et al.;<br>b) Kolt et al.;<br>c) Kolt et al.;<br>d) Kolt et al.;<br>e) Vandelanotte et al.	a) 2018;<br>b) 2017;<br>c) 2017b;<br>d) 2020;<br>e) 2017	Australia	a) The effectiveness of a web 2.0 physical activity intervention in older adults - a randomised controlled trial;<br>b) Using Web 2.0 applications to promote health-related PA: findings from the WALK 2.0 randomised controlled trial;<br>c) Associations between quality of life and duration and frequency of physical activity and sedentary behaviour: Baseline findings<br>from the WALK 2.0 randomised controlled trial;<br>d) Successes and Challenges of an IT- ased Health Behaviour Change Program to Increase Physical Activity;<br>e) Effectiveness of a Web 2.0 Intervention to Increase Physical<br>Activity in Real-World Settings: Randomized Ecological Trial	a) Determine interaction between intervention type and age group or self-efficacy for feasibility outcomes, effectiveness PA;<br>b) Investigated effectiveness of a Web 2.0-based intervention on PA behaviour, and the impact on website usage and engagement;<br>c) Examined the association of HRQoL with PA and sedentary behaviour, using both continuous duration (average daily minutes) and frequency (average daily bouts 10 min) measures;<br>d) Focus on the success factors and challenges in both the RCT and RET;<br>e) Compare usage and effectiveness of Web 1.0 and Web 2.0 intervention in real-world settings	Australians;<br>age 51 (±13) years old;<br>65% female	504

**4**	Al-Eisa et al.	2016	Saudi Arabia	Effect of Motivation by "Instagram" on Adherence to Physical Activity among Female College Students	Investigation of the efficacy of using “Instagram” with a “home-exercise program” as a motivational stimulus in improving PA adherence levels among female college students.	Female college students; age 20.30 (±0.96) years old	47
**5**	Arigo et al.	2015	USA	Addressing barriers to physical activity among women: A feasibility study using social networking-enabled technology	Pilot test an internet-based PA promotion program that combined evidence-based psychoeducation with automated PA self- monitoring and facilitated social connectivity	Women from the general community;<br>age 46 (±13.09) years old;<br>75% caucasian	12 (6 dyads)

**6**	Bopp et al.	2018	USA	Development, Implementation, and Evaluation of Active Lions: A Campaign to Promote Active Travel to a University Campus	Development, implementation, and evaluation of a multistrategy intervention to promote active travel	University students:<br>46% female, 54% male;<br>70% caucasian, and 62% come from the state of Pennsylvania;<br>faculty/staff:<br>age 46.94 (+10.77) years old;<br>51% female, 49% male;<br>primarily caucasian (79%)	610
**7**	Broom et al.	2018	UK	Gotta Catch 'Em All: Impact of Pokémon Go on Physical Activity, Sitting Time, and /ceptions of Physical Activity and Health at Baseline and Three-Month Follow-Up	Examination of differences in PA, sitting time and /ception of PA and health between Pokémon Go users' and non-users' before and after the launch of the game	Pokémon Go users;<br>age 31.4 (+12.1) to 29.6 (+9.0) years old;<br>54 male, 72 female, 1 transgender;<br>predominantly white	127
**8**	Cavallo et al.	2012	USA	A Social Media–Based physical activity Intervention: A Randomized Controlled Trial	Assess efficacy and feasibility of a 12-week PA social support intervention	Female undergraduate students;<br>73% white , 92% non-hispanic	134

**9**	Chee et al.	2014	Malaysia	A Randomised Controlled Trial of a Facebook-based physical activity Intervention for Government Employees with Metabolic Syndrome	Assess the effectiveness of the PA intervention delivered through Facebook	Government employees with metabolic syndrome	124
**10**	Edney et al.	2020	Australia	A Social Networking and Gamified App to Increase Physical Activity: Cluster RCT	Evaluate whether using a gamified app-based PA intervention that connects existing friends via Facebook led to a significant between group difference in objectively measured PA	Australian adults; age 41 ($\pm$12) years old, 74 % female	444
**11**	Elloumi et al.	2018	Netherlands	Exploratory study of a virtual community for physical activity	Exploring differences between a virtual community and a PA monitoring system on PA levels	University staff; age 20-55 years old; 16 female, 13 male	29

**12**	Forrest et al.	2017	Canada	Evaluating social media as a platform for delivering a team-building exercise intervention: A pilot study	Evaluation of the efficacy of an online team building exercise intervention to enhance cohesion, group task satisfaction, physical fitness and programme adherence	First-year university students; age 18.62 (±1.31) years old; 25 female, 2 male	20
**13**	Gotsis et al.	2013	USA	Wellness partners: design and evaluation of a web-based physical activity diary with social gaming features for adults	Exploration of the implementation, evaluation, and effectiveness of a web-based intervention with social networking and social gaming features on participants' PA and anthropometric measures	University staff; age 35.6 years old, 67.6% female, 32.4% male; 18% asian or asian american, 28% hispanic/latino	87

**14**	Greene et al.	2013	USA	The impact of an online social network with wireless monitoring devices on physical activity and weight loss	Assessing the impact of an online social network intervention to increase PA	Adults;<br>age 40+ years old, 60% > 50 years old;<br>78.9% female, 21.1% male;<br>92.8% caucasian, 4.1% Hispanic/Latino, 3.2% other	349
**15**	Irwin et al.	2016	USA	Testing the Efficacy of OurSpace, a Brief, Group Dynamics-Based Physical Activity Intervention: A Randomized Controlled Trial	Efficacy of an online group dynamics-based intervention on PA and group cohesion	young adults;<br>age 19.54 (±1.81) years old;<br>51.1% female, 48.9% male	135

**16**	Joseph et al.	2015	USA	Print versus a culturally-relevant Facebook and text message delivered intervention to promote physical activity in African American women: a randomized pilot trial	Evaluation of a theory-based multi-component intervention using Facebook and text-messages to promote PA among African American women	African American women; age 35.5 (±5.0) years old	29

**17**	Kernot et al.	2019	Australia	Effectiveness of a Facebook-Delivered Physical Activity Intervention for Postpartum Women: A Randomized Controlled Trial	Determination of the effectiveness of a FB app, a team-based, 50-day PA intervention for postpartum women	Postpartum women (up to 12 months); age 31.8 (range: 30.8 to 32.4) years old	120
**18**	Kernot et al.	2014	Australia	Usability Testing and Piloting of the Mums Step It Up Program - A Team-Based Social Networking physical activity Intervention for Women with Young Children	Determination of the usability and effectiveness of a FB app, used to deliver a team based PA intervention for mothers with young children	Mothers with young children; age 34.3 (±2.9) years old	25

**19**	Leinonen et al.	2017	Finland	Feasibility of Gamified Mobile Service Aimed at Physical Activation in Young Men: Population-Based Randomized Controlled Study (MOPO)	Study the feasibility of an automated, gamified, tailored Web-based mobile service aimed at physical and social activation among young men	Young men;<br>age 17.8 (±0.6) to 17.9 (±0.7) years old	354
**20**	a) Looyestyn et al.;<br>b) Edney et al.	a) 2018;<br>b) 2018	Australia	a) A Web-Based, Social Networking Beginners' Running Intervention for Adults Aged 18 to 50 Years Delivered via a Facebook Group: Randomized Controlled Trial;<br>b) Posts, pics, or polls? Which post type generates the greatest engagement in a Facebook physical activity intervention?	a) Determination of the effectiveness of a web-based beginners' running program for adults, delivered via a Facebook group, in increasing PA and cardiorespiratory fitness;<br>b) Determine which post type generates the most engagement and whether engagement was related to PA changes in an intervention delivered via Facebook	Adults;<br>age 35.2 (±10.9) years old;<br>80% female, 20% male	89

**21**	Maher et al.	2015	Australia	A Web-Based, Social Networking Physical Activity Intervention for Insufficiently Active Adults Delivered via Facebook App: Randomized Controlled Trial	Determination of the efficacy, engagement, and feasibility of an online social networking PA intervention with pedometers	Adults;<br>age 18-65 years old;<br>70.9% female, 29.1% male	110
**22**	Mendoza et al.	2017	USA	A Fitbit and Facebook mHealth intervention for promoting physical activity among adolescent and young adult childhood cancer survivors: A pilot study	Testing the feasibility of a mobile health (mHealth) intervention to promote PA	Adolescent or young adult cancer survivors;<br>age 16.6 (±1.5) years old;<br>59.3% female, 40.7 % male;<br>non-hispanic white 71,2%, non-hispanic black 3,4%, hispanic 11,9%, other 13,6%	59
**23**	Nam et al.	2020	Korea	Effects of a social-media-based support on premenstrual syndrome and physical activity among female university students in South Korea	Examination of effects of social-media-based support on premenstrual syndrome (PMS) and PA among female South Korean university students	Menstruating female university students with PMS;<br>age 21.66 (±2.19) to 22.25 (±1.88) years old	64

**24**	Nishiwaki et al.	2017	Japan	A pilot lifestyle intervention study: effects of an intervention using an activity monitor and Twitter on physical activity and body composition	Testing effectiveness of a lifestyle intervention using an activity monitor and Twitter on daily PA and body composition	Healthy adults; age 36 (±3) years old	17
**25**	Petrella et al.	2017	Canada	Hockey Fans in Training: A Pilot Pragmatic Randomized Controlled Trial	Feasability and potential of a real-life intervention with online social network in the follow-up phase to lead to weight loss and related outcomes	Overweight or obese men (BMI values 36.5 (±6.0), corresponding to obese class II); 48.7 (±9.0) years old; 95% white	80
**26**	Pope et al.	2020	USA	Feasibility of smartphone application- and social media-based intervention on college students' health outcomes: A pilot randomized trial	Evaluation of feasibility of combining a mobile app with a theoretically-based, social media-delivered health education intervention to improve college students' health behaviors and outcomes.	College students; age 21.6 years old; 32 female, 12 male; 30 non-hispanic white, 12 asian, 2 non-hispanic black	44

**27**	Pope et al.	2019	USA	Use of Wearable Technology and Social Media to Improve Physical Activity and Dietary Behaviors among College Students: A 12-Week Randomized Pilot Study	Evaluation of feasibility of a theoretically based, socialmedia-delivered health education plus smart watch intervention on improving health outcomes	College students;<br>age 21.2 (±4.0) to 21.8 (±2.8) years old;<br>28 female, 10 male;<br>27 non-hispanic white, 11 asian	38
**28**	Pope et al.	2018	USA	Feasibility of smartphone application and social media intervention on breast cancer survivors' health outcomes	Investigation of the feasibility of a social media-based health education intervention in combination with a mobile health app to improve breast cancer survivor PA and health.	Female breast cancer survivors;<br>age 45.8 (±10.2) years old;<br>9 caucasian, 1 asian	10

**29**	Pope et al.	2018_b	USA	Effectiveness of Combined Smartwatch and Social Media Intervention on Breast Cancer Survivor Health Outcomes: A 10-Week Pilot Randomized Trial	Evaluation of the effectiveness of a combined smartwatch and social media-based health education intervention on breast cancer survivors' health outcomes	Female breast cancer survivors; 50.6 (±7.4) to 54.9 (±11.0) years old; 29 caucasian, 1 asian	20
**30**	Rote et al.	2015	USA	The Efficacy of a Walking Intervention Using Social Media to Increase Physical Activity: A Randomized Trial	Examination of the efficacy of a Facebook Social Support Group to increase PA.	Female college freshmen; age 18.6 (±0.7) years old; 77.8% white, 11.1% black, 6.3% asian, 4.8% hispanic	63

**31**	Rovniak et al.	2016	USA	Engineering Online and In-/son Social Networks for Physical Activity: A Randomized Trial	Assessment of the contributions of different procedures for building social networks on health-outcomes, by conducting a RCT based on the Social Networks for Activity Promotion model.	Adults;<br>age 50.3 (+8.3) years old;<br>61.7% female, 38.3% male,<br>91.9% non-hispanic white	308
**32**	Ruotsalainen et al.	2015	Finland	Effectiveness of Facebook-Delivered Lifestyle Counselling and Physical Activity Self-Monitoring on Physical Activity and Body Mass Index in Overweight and Obese Adolescents: A Randomized Controlled Trial	Evaluation of the effects of a Facebook-delivered lifestyle counselling intervention on PA and BMI	Overweight and obese adolescents;<br>age 14.7 (+0.8) years old;<br>70% female, 30% male	44

**33**	Schneider et al.	2015	USA	An Online Social Network to Increase Walking in Dog Owners: A Randomized Trial	Examination of a social networking website (Meetup™) as a tool to deliver a multi-component dog walking intervention to increase PA	Dog owners from two neighborhoods;<br>age 47.49 (+12.26) to 49.20 (+13.72) years old;<br>76.47% female, 23.53% male;<br>84.31% non-hispanic-white, 15.69% hispanic or other	102
**34**	Schoenfelder et al.	2017	USA	Piloting a mobile health intervention to increase physical activity for adolescents with ADHD	Evaluation of feasibility, acceptability, and preliminary efficacy of a behavioral intervention to increase daily habitual PA for adolescents with ADHD.	Adolescents with ADHD;<br>age 15.5 (+1.4) years old;<br>54% female;<br>80% caucasian,10% asian american, 10% multi-racial	11

**35**	Todorovic et al.	2019	Serbia	Can social media intervention improve physical activity of medical students?	Assesment and improvement of PA-levels in medical students through a social media intervention	First- (61.6%) and fifth-year (38.4%) medical students; 73% female, 27% male	375
**36**	Tong et al.	2019	Australia	Efficacy of a Mobile Social Networking Intervention in Promoting Physical Activity: Quasi-Ex/imental Study	Exploration of engagment, usability and efficacy of a social networking mobile app plus wearable tracker as part of an intervention to improve PA.	76% university students; age 23.6 (±4.6) years old; 51% female	52
**37**	Torquati et al.	2018	Australia	Changing Diet and Physical Activity in Nurses: A Pilot Study and Process Evaluation Highlighting Challenges in Workplace Health Promotion	Evaluation of key implementation and context factors of a diet and PA workplace intervention, using the Reach, Effectiveness, Adoption, Implementation, and Maintenance framework	Nurses; age 41 (±12) years old; 87% female	47

**38**	a) Valle et al.;<br>b) Valle et al.;<br>c) Valle et al.	a) 2013;<br>b) 2015;<br>c) 2017	USA	a) A Randomized Trial of a Facebook-based physical activity Intervention for Young Adult Cancer Survivors;<br>b) Exploring Mediators of PA in Young Adult Cancer Survivors: Evidence from a Randomized Trial of a Facebook-Based Physical Activity Intervention;<br>c) Engagement of young adult cancer survivors within a Facebook-based physical activity intervention	a) Evaluation of the feasibility and preliminary efficacy of a Facebook-based intervention, aimed at increasing MVPA, compared to a Facebook-based self-help comparison condition; b) Examination of the effects of a PA intervention for young adult cancer survivors on changes in self-efficacy, social support, and self-monitoring, determination whether changes in these social cognitive theory constructs mediated the relationship between the intervention and changes in PA;<br>c) Characterization of Facebook engagement by young adult cancer survivors in the context of a PA intervention program	Young adult cancer survivors;<br>age 31.7 (±5.1) years old;<br>91% female;<br>91% non-hispanic white race, 9% black/other	86

**38**	Van Woudenberg et al.	2020	Netherlands	Testing a Social Network Intervention Using Vlogs to Promote Physical Activity Among Adolescents: A Randomized Controlled Trial	Effectiveness of social network intervention in comparison to mass media or no intervention	Pupils; age 11.35 (+1.34) years old; 53% female, 47% male	446
**40**	Wang et al.	2015	Singapore	Use of facebook in physical activity intervention programme: A test of self-determination theory	Examination of changes to activity level and psychological consequences of incorporating social network interactions into a PA intervention programme using the self-determination theory framework	Undergraduate students; age 22.3 (+1.51) years old; equal gender distribution	62

**41**	Wang et al.	2017	China	Short-term effects of social encouragement on exercise behavior: insights from China's Wanbu network	Exploration of the short-term effects of social encouragement on exercise behavior in China	No information on the study population was collected	5.010
**42**	Wójcicki et al.	2014	USA	Promoting Physical Activity in Low-Active Adolescents via Facebook: A Pilot Randomized Controlled Trial to Test Feasibility	Examination of the feasibility and efficacy of using Facebook for the delivery of a PA intervention	Adolescents;<br>age 13.48 years old;<br>52% female, 48% male;<br>62% white, 5% black, 9% asian, 24% biracial, 9% latino	20

**43**	Xian et al.	2017	USA	An Initial Evaluation of the Impact of Pokémon GO on Physical Activity	Examination of the impact of Pokémon GO on PA	Adults;<br>median age 25 (21-29 IQR) years old;<br>47.9% female	167
**44**	Zhang et al.	2016	USA	Support or competition? How online social networks increase physical activity: A randomized controlled trial	Comparison of supportive or competitive relationships within social networks to increase PA	Graduate and professional students;<br>age 25.2 (±3.4) years old;<br>74,1% female, 25,9% male;<br>44,6% white, 7.3% black, 7.8% hispanic, 36.3% asian	790

**45**	Zhang et al.	2015	USA	Efficacy and causal mechanism of an online social media intervention to increase physical activity: Results of a randomized controlled trial	Identification of social media features that can increase PA	Graduate students; age 25.8 (+4.0) years old; 29% male	217

PA= physical activity; AT= active travel; PMS= premenstrual syndrome; ADHD= attention deficit hyperactivity disorder; MVPA= moderate to vigorous physical activity; BMI= body mass index; BP= blood pressure: HRQoL

h. Study design	i. Study arms	j. Type of intervention	k. Duration of intervention	l. Social media platform used	m. PA outcomes	n. Secondary health outcomes
RCT	I1= Web 1.0;<br>I2= Web 2.0;<br>C= Control group	WALK 2.0:<br>I1= 10,000 Steps website with standard Web 1.0 features (data entry, text forum submissions, public forum) + pedometer;<br>I2= WALK 2.0 website with Web 2.0 features ('status updates', streams, blogs, internal emails, and forum posts);<br>C= Pa/-based logbook	18 months	Study-specific website with social network features	a)<br>1. Minutes MVPA, steps/day;<br>e)<br>2. Total PA (% min/week)	c)<br>3. HRQoL;<br>e)<br>4. BMI,<br>5. Quality of life

Quasi-ex/imental study	I= Intervention group; C= Control group	I= Instagram-study account (educational posts about PA, reminders for the exercise sessions with link for freely available YouTube exercise video, encouragement to post pictures of adherence sheet for motivation of others; C= Link to YouTube exercise video provided at the beginning of the study	4 weeks	Instagram, YouTube	1. Adherence to exercise sessions (completers of 8 or more sessions were defined as adherent)	2. Motivational factors
Pilot study	I= Intervention group	I= Webinar + website with worksheets for PA goal setting / dyad + FitBit + private group at Website	4 weeks	Fitbit	1. PA goals, PA	

Pilot study	I= Intervention group	I= "Active Lions" campaign targeting behavioral beliefs, social support, and time constraints in relation to active travel (=AT), tailored smartphone app for adoption and maintenance of AT, social marketing online/on campus and via social media targeting attitudes and beliefs about PA	1 year	Facebook, Twitter	1. AT to campus (trips walking, biking), observations of AT participation onto campus	
Observational study	No randomization, comparison of three groups: I1= Users of Pokémon Go; I2= Users that became non-users; C= Non-users	I= "Pokémon GO", a location-based augmented reality game, progress in the game is achieved through real-life PA (eg, walk, bike, or drive), participants were surveyed immediately after the launch of the game and again 3 months later	3 months	Pokémon GO	1. Days and minutes of vigorous and moderate PA and walking, sitting on weekdays and weekends	2. BMI
RCT	I= Intervention group; C= Control group	I= INSHAPE, online social network + self-monitoring + website with educational information; C= Education-only control	12 weeks	Facebook	1.PA	2. Perceived social support for PA

RCT	I= Intervention group;<br>C= Control group	I= Facebook group, information materials, comments, posts, step logging;<br>C= Group meetings, step logging	6 months (4 months intervention, 2 months follow-up phase)	Facebook	1. Steps/day	2. Components of metabolic syndrome (weight, height, BMI, waist circumference, hip circumference, waist to hip ratio, body fat ,blood pressure, HDL, LDL triglycerides, cholesterol, fasting glucose)
RCT	I1= Gamified app group;<br>I2= Basic app group;<br>C= Control group	I1= "Active Team" app and a wrist-worn pedometer, connected with Facebook profile, steps count and competition within teams;<br>I2= " Active Team" self-monitoring features and a wristworn pedometer;<br>C= Waitlist control	100 days	Study-specific app (Active Team), Facebook	1. Daily minutes of MVPA, self-reported PA	3. HRQoL<br>4. Symptoms of depression, anxiety and stress<br>5. Psychological well-being
Pilot study	I= Intervention group;<br>C= Control group	I= "TogetherActive V2" system, online portal used in teams, self-monitoring, individual and group comparison and goal-setting, plus Fitbit;<br>C= Fitbit + app for self-monitoring without community functionalities	9 weeks	Study-specific virtual community (TogetherActive V2 system)	1. Steps/day, Steps/hour, Steps/week	

Pilot study	I= Intervention group	I= Participants were divided into four fitness groups led by 1 of four fitness leader: all received a standardised exercise programme "Freshman Fit-Teen" (biweekly in-/son sessions) + online team-building exercise protocol (via Facebook, led and delivered by the specifically trained leaders through 10-15 online sessions following the exercise classes, messages could also be seen afterwards on the group wall)	8 weeks	Facebook	1. Physical fitness (successful repetitions of pushups, partial curl-ups and vertical jump height; successful repetitions, height in inches)	
RCT	I1= PA diary + game first, than only PA diary;<br>I2= PA diary first, than PA diary + game	"Wellness Partners" program: participants ("egos") invited other member of their social network into their team ("alters"), participants changed condition after first follow-up:<br>I1= website + PA diary (logging and sharing PA, private messages) + social gaming (creation of virtual character, progression of character through rewards for PA, comparison with other's characters) I2= [illegible]	13 weeks	Study-specific website	1. Exercise frequency in min/day & days/week	2. BMI (only "egos")<br>3. Fat mass (only "egos")<br>4. Fat /centage (only "egos")

RCT	I= Intervention group; C= Control group	I= Printed lifestyle guidelines on diet and exercise plus "iWell" online social network for goal setting, monitoring and sharing steps and weight, accelerometer; C= Printed materials only	6 months	Study-specific social network (iWell)	1. Min/week overall PA, leisure time walking	2. Weight<br>3.Cholesterol<br>4. Triglycerides
RCT	I1= Social support; I2= Group dynamics-based low presence; I3= Group dynamics-based high presence; C= Individual	Repeated plank challenge, interrupted by app and social network usage:<br>I1= App for interaction and exchange of ex/iences;<br>I2= I1 plus team-building component and joint goal-setting;<br>I3= I1 and I2 plus /forming the challenge together via video;<br>C= No contact, individual challenge	one day	Study-specific app (OurSpace)	1. Amount of time (seconds) in plank position	2. /ceptions of group cohesion<br>3. Rating of /ceived excertion

RCT	I1= Facebook intervention; I2= Print-based intervention	I1= Pedometer Facebook group (weekly, culturally tailored, posts for PA promotion, motivational text messages, discussion prompts, self-monitoring and goal-setting) + Emails; I2= Emails (feeback & encouragment) + fortnightly mailings of self-help booklets (information and promotion of PA and health, tips and strategies to increase daily PA up to a minimum of 150 min/week of MVPA, researchers set a static goal of 8,000-10,000 steps each day	8 weeks	Facebook	1. Sedentary behavior, light PA and MVPA, step counts	2. Social cognitive theory variables (self-efficacy for PA, social support for exercise, self-regulation and outcome expactations for PA) 3. BMI

RCT	I1= "Mums Step it Up" condition;<br>I2= Pedometer condition;<br>C= Control group	I1= "Mums Step it Up" program (participants served as "captains" of self-recruited teams out of existing friend groups): pedometer + Facebook app (tracking /monitoring of own and team steps, short-term and long-term goal-setting, comparison within and with other teams, interaction, challenges, feedback, virtual gifts and awards);<br>I2= Pedometer + log book for self-monitoring (for a /iod of 50 days);<br>C= Written advice on increasing PA	6 weeks (follow-up after 6 months)	Study-specific app (Mums Step it Up), Facebook	1.Time spent walking, accelerometer derived and self-reported MVPA, total activity count	2. Sleep quality/quantity<br>3. Depressive symptoms (common for post-partum) 4. QoL<br>5. BMI
Pilot study	I= Intervention group	I= "Mums Step it Up" program (participants served as "captains" of self-recruited teams out of existing friend groups): pedometer + weekly e-mails (reminders and feedback) + Facebook app (daily tips for increasing PA, peer encouragement and support, goal setting, feedback, virtual gifts & awards)	4 weeks	Study-specific app (Mums Step it Up), Facebook	1. Walking time, time spent with moderate or vigorous PA, steps/week	

RCT	I= Intervention group;<br>C= Control group	I= PA monitor and gamified app "MOPOrtal" (tailored health information, exercise/PA instructions, feedback, social networking with competition, conflict, collaboration, scoring /rewards for PA);<br>C= Received a blinded PA monitor	6 months	Study specific app (MOPOrtal) with social networking features	1. MVPA	2. Height<br>3. Waist circumference<br>4. Grip strength<br>5. Weight<br>6. Body composition<br>7. Aerobic fitness
RCT	I= Intervention group;<br>C= Control group	I= "UniSA Run Free", a beginners running program, delivered via FB group (additional daily posts, including information and encouraging social interaction);<br>C= Self-directed running program, structure of "UniSA Run Free" was provided	8 weeks	Facebook	a)<br>1. MVPA<br>b)<br>2. Interaction between Facebook engagement and compliance with the running program	a)<br>3. Cardiorespiratory fitness<br>4. Social-cognitive theory constructs (self-efficacy, barriers to exercise, attitudes towards exercise, amount of social support regarding PA)

RCT	I= Intervention group; C= Control group	I= Pedometer + Facebook app "Active Team" (daily tips for PA, team building for encouragement and support (consisting of pre-existing Facebook friends), goal-setting & awards/gifts); C= Waitlist control group	50 days	Study-specific app (Active Team), Facebook	1. Overall MVPA, types of PA se/ately (weekly walking time, other moderate PA, and vigorous PA), interaction between MVPA and intervention dose	2. QoL
Pilot RCT	I= Intervention group; C= Control group	I= Fitbit (accelerometer + mHealth app) + social network for interaction and delivery of motivational messages; C= Standard care	10 weeks	Facebook	1. MVPA, sedentary time	2. QoL<br>3. Self-determination theory (SDT) constructs (motivation for PA , psychological needs satisfaction)
RCT	I= Intervention group; C= Control group	I= Text-messages, e-mails and social-media based support for PMS and PA (information & education, sharing of ex/iences & coping strategies, feedback & support); C= Received information on PMS and PA	one menstrual cycle (about 28 days)	Fitbit Flex app with social networking feature	1. MET (min/ week)	2. PMS symptoms and levels (low mood/loss of pleasure, endogenous depressive features, liability, atypical depressive features, hysteroid features, hostility/anger, social withdrawal, anxiety, increased well-being, impulsivity, organic mental features, signs of water retention, general physical discomfort, autonomic physical changes, fatigue, impaired social functioning, miscellaneous mood/behaviour changes, miscellaneous physical changes)

Pilot study	I= Twitter group;<br>C= Normal group	I= Activity monitor + encouraged to walk >10,000 steps/day + feedback from observer + Twitter to send daily tweet; C= Same as T without Twitter account	6 weeks	Twitter	1.Number of steps, amount of total PA	2. Body composition and blood pressure (BP)
Pilot RCT	I= Intervention group;<br>C= Control group	I= "Hockey FIT" program, active phase (educational and exercise classes, pedometer-based walking program) followed by minimally supported phase (app and social network to encourage maintenance, pedometer);<br>C= Waitlist control	12 weeks, followed by minimaly supported 40 weeks	Study-specific app and social network (Hockey FIT)	1. Steps/day,<br>total PA,<br>sedentary time	2. Weight loss<br>3. BMI<br>4. Waist circumference (cm)<br>5. Resting systolic blood pressure (mmHg)<br>6. Resting diastolic blood pressure (mmHg)<br>7. Eating beahvior<br>8. Alcohol consumption<br>9. Psychological and HRQoL
Pilot RCT	I= Intervention group;<br>C= Control group	I= App "MapMyFitness" (logging & tracking of PA) + Facebook group (health-education tips to promote awareness of PA-related behaviors, encouragement for implementation and posting to the group);<br>C= Content identical Facebook group, no app	10 weeks	Facebook	1. Sedentary behavior, MVPA	2.Height<br>3. Weight<br>4. Body fat /centage<br>5. Cardiorespiratory fitness<br>6. PA-related self-efficacy<br>7. Social support<br>8. Enjoyment<br>9. Barriers<br>10. Outcome expectancy

Pilot RCT	I= Intervention group;<br>C= Control group	I= Smartwatch (logging/trackin PA) + Facebook group (twice-weekly health-education tips related to PA and nutrition);<br>C= Content identical Facebook group, no smartwatch	12 weeks	Facebook	1. Steps/minute, categorized into sedentary behaviour, light PA (LPA) and MVPA	2.Height<br>3. Weight<br>4. Body fat /centage<br>5. Cardiorespiratory fitness<br>PA-related self-efficacy<br>7. Social support<br>8. Enjoyment<br>9. Barriers<br>10. Outcome expectancy<br>11. Intrinsic motivation<br>12. Dietary behaviour	6.
Pilot study	I= Intervention group	I= App "MapMyFitness" (logging & tracking of PA, goal setting) + Facebook group (twice-weekly health-education tips related to PA)	10 weeks	Facebook	1. Mean daily duration of sedentary behavior,<br>light PA & MVPA,<br>steps/day,<br>energy expenditure in kcal	2.Height<br>3. Weight<br>4. Body fat /centage<br>5. Cardiorespiratory fitness<br>PA-related self-efficacy<br>7. Social support<br>8. Enjoyment<br>9. PA related barriers<br>10. Outcome expectancy<br>11. QoL	6.

RCT	I= Intervention group;<br>C= Control group	I= Smartwatch (logging/trackin PA) + Facebook group (twice-weekly health-education tips related to PA);<br>C= Content identical Facebook group, no smartwatch	10 weeks	Facebook	1. Mean daily duration of sedentary behavior, light PA & MVPA, steps/day, energy expenditure in kcal	2.Height<br>3. Weight<br>4. Body fat /centage<br>5. Cardiorespiratory fitness 6.<br>PA-related self-efficacy<br>7. Social support<br>8. Enjoyment<br>9. Barriers<br>10. Outcome expectancy<br>11. QoL
RCT	I= Intervention group;<br>C= Control group	I= Standard walking intervention (pedometer + pa/ walking log, /sonalized weekly step goals) + Facebook Social Support group (discussion, feedback & encouragement, educational posts about PA); C= Standard Walking Intervention + weekly e-mails with same content on PA as I	8 weeks	Facebook	1. Steps/day	2. Height<br>3. Weight<br>4.Waist circumference

RCT	I1= Activity group;<br>I2= Social networking group;<br>I3= Promotion group	Weekly Emails for promotion and information on walking and other PA: I1= + 12-week evidence-based online fitness walking program (introductory in-/son walking session, feedback, goal-setting, pedometer & walking log);<br>I2= Same as I1 + online social network (self-organization of walking sessions with other participants, discussion team, feedback & comparison);<br>I3= Only weekly Emails	12 weeks	Ning Networks, Facebook	1. MVPA	2. Aerobic power (VO2max)<br>3. BMI<br>4. Waist circumference	
RCT	I1= Facebook counselling;<br>I2= Facebook counselling + PA self-monitoring;<br>C= Control group	I1= Se/ate Facebook groups for adolescents and parents (lifestyle-counselling regarding PA, tailored exercise programs, discussions and communication);<br>I2= Same as I1 + accelerometer;<br>C= Counselling from the school nurse, if required	12 weeks	Facebook	1. Objectively: sedentary time or very light PA, light, moderate, vigorous, and 'vigorous plus' PA;<br>Self-reported:<br>total PA,<br>screen time	2. BMI	

RCT	I= Meetup condition; C= AHA condition	I= Introductory in-/son meeting with the other participants + Meet-up groups (message boards for interaction between participants, invitations for joint neighborhood walks led by a staff member, promotion of dog-friendly community events, informational newsletter regarding dog-walking) + activity monitor; C= monthly emails with encouragement to begin walking and a link from the AHA (American Heart Association's PA website) website for starting a PA program	6 months	Meetup	1. Steps/day	2. Dog and PA tool (weekly dog walking minutes, dog walking barriers, /ceived outcomes from dog walking<br>3. Social support for walking (whether friends/family support the walking exercise behaviour)<br>4. Sense of community (community membership, influence, whether or not one's needs are being met and presence of a shared emotional connection within one's community)
Pilot study	I= Intervention group	I= Fitbit + Facebook group (challenges, rewards for goal reaching and social interaction, sharing of steps and feedback) + daily messages	4 weeks	Facebook	1. Steps/day	2. ADHD symptoms (teen- and parent-reported)<br>3. Mood valence

RCT	I= Intervention group;<br>C= Control group	I= Facebook group (motivational posts for PA: pictures, texts, and discussions);<br>C= nothing	1 month	Facebook	1. MET/week = number of days with PA*duration in minutes*k (categorized into sufficient/insufficient PA, sufficient PA: >600 MET-min/week)	
Pilot study	I= Intervention group	I= Mobile app ("fit.healthy.me" for comparison of step counts, following others, social support), + FitBit (self-monitoring and logging steps) + and SMS/Emails (prompts, cues, and reminders)	6 months	Study-specific app (fit.healthy.me) and social network	1. Steps/day	
Pilot study	I= Intervention group	I= Pedometer + smartphone app for goal setting regarding diet and PA + private Facebook group for social support	3 months	Facebook	1. Sedentary behavior, light activity,<br>MVPA (min),<br>steps/day	2. Dietary patterns<br>3. Weight<br>4. Height<br>5. BMI<br>6. Waist circumference<br>7. Blood pressure<br>8. Diet and PA self-efficacy/social support<br>9. Self-rated health

RCT	I= Intervention group; C= Control group	I= "FITNET" program: pedometer (with linked website for logging & tracking, goal-setting, feedback) + Facebook-based group (expanded behavioral lessons/strategies about PA, provision of links to websites related to PA and/or cancer survivorship, moderated posts to encourage support/discussion); C= pedometer + Facebook-based self-help group with basic information about PA, provision of links to websites related to PA and/or cancer survivorship	12 weeks	Facebook	a)<br>1.MVPA, strenuous, moderate and mild intensity exercise, interaction of PA with adherence and acceptability<br>b)<br>2. Interaction of PA outcomes with secondary health outcomes (SCT-constructs)<br>c)<br>3. Interaction of PA outcomes with engagement	a)<br>4. QoL<br>5. Height<br>6. Weight<br>b)<br>7. Self-efficacy (rating of confidence to being able to stick to different PA goals)<br>8. Social support (directly related to exercise, by friends or family in real life or on social media)<br>9. Self-monitoring (PA and form of self-monitoring)

RCT	I1= Social network intervention<br>I2= Mass media intervention<br>C= Control group	I1= Vlogs filmed by acquainted peers to implement a social norm of being physically active, distributed via social network ("MyMovez App")<br>I2: Mass-media intervention via social network, vlogs from unfamiliar peers<br>C: Short videos unrelated to PA	5 months	Study-specific app and social network (MyMovez App)	1. Steps/day, intensity of activity	2. Sociometric status<br>3. PA-related social norm<br>4. Enjoyment<br>5. Self-efficacy<br>6. Motivation
RCT	I1= 3 hours fitness class;<br>I2= 3 hours fitness class + Facebook;<br>I3= 1 hour fitness class + Facebook; C= Control group	I1= Weekly 3hour face-to-face fitness class as part of an university course (education, exercise, practising);<br>I2= Identical fitness classes + Facebook group (informational and discussion posts, sharing of /sonal ex/iences);<br>I3= Voluntary 1 hour fitness classes (exercise) + identical Facebook group;<br>C= No intervention	8 weeks	Facebook	1. Frequency/duration of PA in last week, MVPA, (categories: < 1000 MET-min/week = 1; 1000 to 1999 METmin/week= 2; 2000 to 2999 MET-min/week= 3; > 3000 MET-min/week= 4)	2. Intrinsic motivation inventory (/ceived autonomy, /ceived competence, relatedness, /ceived enjoyment and vitality) 3. Subjective vitality

Observational study	I= Intervention group	I= Wrist strap (recording and uploading steps/day) + social network "Wanbu" (summary and visualization of the data, exchanging information with friends and interaction through liking, commenting and sharing)	Data was collected for 18 months	Wanbu network	1. Exercise level, steps/day, relationship between exercise level and social encouragement
Pilot RCT	I= Behavioral condition; C= Informational condition	SMART-Trial:<br>I= Facebook group with PA-related information and promotion + individual Facebook messages with behavioral modules (YouTube videos);<br>C= Access to Facebook group without behavioral modules	8 weeks	Facebook	1. Steps/day, categorized into sedentary, light, moderate, and vigorous activities,<br>self-reported MVPA, sedentary behavior

Observational study	I= Intervention group	I= "Pokémon GO", a location-based augmented reality game, progress in the game is achieved through real-life PA (e.g., walk, bike, or drive), participants retrospectively uploaded screenshots of the “Pokémon GO Trainer Profile” and all recorded daily steps displayed on the iPhone Health dashboard 3 weeks before and 3 weeks after the Pokémon GO release date	7 weeks	Pokémon GO	1. Steps/day, /centage of days >10.000 steps/day, relationship between dose (engagement) and daily steps
RCT	I1= Social support; I2= Social comparison; I3= Social support + comparison; C= Control group	"SHAPE-UP" program, exercise classes + SHAPE-UP website: I1= Website plus social network for interaction and team building; I2= Website plus ranking device, no social interaction; I3= Components of I1 and I2 combined; C= Access to website, registration for exercise classes, no social component	11 weeks	Study-specific website and social network (SHAPE-UP)	1. Number of attended exercise classes

RCT	I1= Media condition; I2= Social condition; C= Control group	"SHAPE-UP" program, exercise classes + SHAPE-UP website: I1= Website for registration and information on exercise classes + motivational messages; I2= Website plus social network (comparison but no communication); C= Website only	13 weeks	Study-specific website and social network (SHAPE-UP)	1. Number of attended exercise classes, self-reported days with moderate or intensive or strength-building exercises

_= health-related quality of life; QoL= quality of life

o. Outcomes acceptability, use, usability	p. Results effectiveness on PA	q. Results secondary health outcomes	r. Results acceptability, use and usability
a)<br>7. Internet self-efficacy<br>8. Usability<br>9. Satisfaction<br>10. Website usage;<br>b)<br>11. Engagement;<br>d)<br>12. Feasibility<br>13. Lessons learned;<br>e)<br>14. Website engagement and retention<br>15. Website usability	a)<br>1. I1 more effective than the logbook at 3 months, effect was significantly stronger in older compared to younger adults OR = 0.23 (95% CI=0.06- 0.87), p < .05<br>e)<br>2. I1 increased PA by 92.8 minutes/ week more compared to I2, p=.005, Cohen d effect size = 0.29,at baseline 57 % of participants in both groups engaged in sufficient PA, at 3-month 77% of I1 participants and 71.5% of I2 participants engaged in sufficient PA	c)<br>3. Significant positive association between PA duration and general HRQoL (path coefficient = 0.294, p<0.05); people with more average PA and less daily sedentary behaviour had higher HRQoL scores (path coefficient = -0.217, p<0.05);<br>e)<br>4.Reduced over time in I1 and I2 (ITT analysis for Web 2.0: change BMI=-1.03 kg/m2, p=.002; completer analysis for Web 1.0: change in BMI=-0.58 kg/m2, p=.002)<br>5. No significant effects, except for physical functioning, improvement (3.6 units) in I1 compared to I2, p<.001	a)<br>7. Higher Internet self-efficacy (younger adults) was associated with more time spent on the website, and significantly stronger for I1 at 3 months (OR = 1.11, 95% CI = 1.00–1.51) p = .05<br>8. No significant differences<br>9. Older adults less likely to have a high satisfaction of the interventions compared to younger (OR = 0.23, 95% CI = 0.06- 0.87), p < .05<br>10. Time spent on the website I1 compared to I2 intervention from baseline to 3 months was significantly higher for older compared to younger adults (OR = 1.47, 95% CI= 1.01-2.14), p < .05<br>11. No significant differences<br>d)<br>12.Total number of website visits + average number of website visits significantly higher in the I1 than I2 (p = 0.002), as was the time between first and last visit to the website (p = 0.001).<br>13. Challenges regarding intervention development within a research context, establishing a self-sustaining community, rapid pace of change in web-based technology and trial design implications, outcome measures for ecological trials, engagement, non-usage and study attrition in realworld trials were encountered; e)<br>14. total number of visits 9.0 (±19.3) vs 5.7 (±17.9), p=.002 and average number of visits 0.7 (±1.6) vs 0.4(±1.4), p=.002 higher in I1 as well as time between first and last visit 3.6 (±3.7) vs 2.2 (±2.9), p<.001, total days 0.7 (±5.9) vs 0.2 (±1.5), p=.03 and average days 0.05 (±0.45) vs 0.01 (±0.10), p=.02 with a step entry comment higher in I1, 21.99% (292/1328) of participants will use website after 2 weeks and 6.55% (87/1328) after 10 weeks<br>15. I1 participants rated usefulness of features more highly compared to I2 participants, many did not use some of the interactive features or indicate that they were very useful

3. Program feedback (flexibility, effectiveness, and motivation level for given program activity)	1. Higher adherence in I (17%) compared to C (4%), p=0.04	2. 47 % of the students were motivated to gain positive health and 2% by social recognition and health pressure	3. 72% found the program flexible, 53% effective, and 64% felt motivated by it; reasons for non-adherence were academic stress (42.5%), not enough time (25.5%), Program is hard to /form (8.5%), not motivated by program (2.1%), or others (21.3%)
3. Partner communication<br>4. FitBit use<br>5. Treatment response and feedback<br>Social comparison	1. All participants met all or some of their goals (e.g. step goal but not MVPA goal)<br>6. during week 1 and 2, 73% during week 3 and 82% during week 4 ;<br>participants' starting activity levels averaged 5995 steps/day (+3956) and 32.38 minutes of MVPA/week (+27.64) ; participants' highest PA weeks reached averages of 10,686 steps / day (+3168) and 51 total minutes of MVPA (+29.74)		3. On average, participants posted eight times(±4.09) or 2/week<br>4. FitBit reports showed 93% compliance with program recommendations for daily wear (range 68-100%)<br>5. Participants' average responses demonstrated: M=3.45 of 5 found the program effective;<br>M=3.45 of 5 were satisfied with the program's approach to increasing PA;<br>M=3.90 of 5 were confident with the ability to maintain their PA gains;<br>M=3.36 of 4 indicated that they would recommend the program to another woman interested in increasing PA;<br>6. Those who endorsed stronger overall tendencies toward comparison at baseline were more likely to report viewing their leaderboards throughout the program (t=2.08, p=0.06, d=1.20); individuals who endorsed stronger tendencies toward upward comparison reached higher peak MVPA minutes during the program (r=0.64, p=0.03)

2. App evaluation<br>3. Social marketing evaluation 4.<br>Social media evaluation	1. Students: 64.23 (+ 42.71) % of trips as active travel compared to 49.19 (+ 40.34) pre-intervention, p=.001;<br>faculty staff: no significant changes,<br>Staff & students: followers on social media had higher increase 51.93 (+46.61) trips as AT compared to those who were not followers 27.46 (+ 41.66), p=.02;<br>observations: n= 2891 walkers and n= 372 bikers were observed at follow-up, compared to n= 1377 walkers and n= 219 bikers		2. 510 downloads after 1 month,resulting in 2002 sessions, 16.681 page views, with 8.33 pages/ session 3. 2570 clicks on online advertisments (click through rate 0.14%); online stories targeting students generated 181 reads, and those targeting employees generated 97 reads; users of the app participated in 11 on-campus events 4. 177 Facebook followers and 103 Twitter followers over the year, highest enagement for educational and interactive postings
3. Feedback<br>4. Adherence	1. Days and minutes of vigorous PA: less for I1 compared to I2 and C, p<0.05;<br>minutes of walking: more for I1 compared to I2 and C, p<0.05; sitting time on a weekday: within group increase for I1, p<0.05; sitting time on weekends: within group increase for I2, p<0.05	2. No significant group differences	3. I1 considered the benefits of Pokémon Go for improving health higher compared to I2 and C, p< 0.05<br>4. 18% of participants continued to use Pokémon Go up to follow-up, 56% were non-users at T0 and at T1 and 26% were user' that became non-users
3. Facebook engagement	1. No significant differences in PA and perceived social support	2. No significant differences in perceived social support	3. I logged into the INSHAPE website on average approximately every 2 weeks vs. 2 x over the course of the intervention among C (584 total logins);<br>both website logins and Facebook activity declined during the intervention

	1. Both groups increased their number of steps from baseline to first phase, I had a significantly greater increase in the number of steps / day than C	2. Increase in the number of steps/day was strongly associated with an increase in HDL cholesterol (r = 0.745(, p<0.001 and a decrease in systolic blood pressure (r = -0.965) p<0.001, diastolic blood pressure (r=-0.930) p<0.001, triglycerides (r=-0.811) p< 0.001, waist circumference (r=-0.968) p<0.001 and fasting glucose (r=-0.819) p<0.001	
6. Engagement<br>7. App usability	1. No significant differences<br>2. I1 reported completing 78 more minutes of MVPA/week at 9 months than the I2, 147 more minutes than C (F=3.1), p=0.02	3. No significant changes<br>4. No significant changes<br>5. No significant changes	6. Of 100 days, participants logged steps for an average of 72 (±35) days in I1 and 67 (±32) days in I2 7. I1 reported an average System Usability Scale score of 66 (±15), indicating that the app was generally regarded as being of average quality
2. Usability<br>3. Engagement	1. Steps / hour: I shows 37% activity and 8% low activity, compared to 35% activity and 11% low activity for C;<br>steps / week: I no decrease in PA compared to C, considerable increase in I compared to baseline at week 9 (897 steps in active hours) compared to C (805 steps in active hours)		2. Minor improvements in usability compared to an earlier version of the program<br>3. No usage of the messaging function, I accessed the portal about twice as often as C (approximately 430 sessions for I and 200 for C)

2. Possible determinants of adherence or dropout 3.Evaluation of the online TB exercise intervention	1. Push-ups: increase from 27.5 (±13.48) to 36.93 (±12.36) repetitions, $p< 0.01$; vertical jump height: increase from 29.47 (±7.37) to 34.20 (±10.27) inches, $p<0.01$		2. ATG-T subscale of cohesion: improvement from 7.49 (+ 1.05) to 7.87 (+ 0.88) points, $p= 0.04$;<br>most frequently coded explanation for completion was a sense of cohesion, if participants completed the online TB exercise protocol (implementation phase) they were more likely to adhere to the exercise programme<br>3. Group task satisfaction: improvement from 6.03 (+ 0.77) to 6.31 (+ 0.65) points, $p= 0.02$
	1. Both groups increased number of self-reported days of exercise/<br>week	2.Decrease at first follow-up (-0.19), $p=0.02$, but no maintenance to second follow-up<br>3. No significant differences<br>4. No significant differences	

5. Number of messages sent via social network 6. Number of accelerometer uploads	1. Leisure time walking: 164% increase for I (341 min/week), compared with a 47% increase for C (208.6 min/week), $p<.01$	2. Greater for I (5.2 pounds) compared to C (1.6 pounds), $p< 0.01$<br>3. No significant differences<br>4.Reduction at 3 months in I (102.5) compared to C (111.0), $p<.05$, but difference was not sustained up to post-intervention	5. Number of messages sent via social network: positively related to increases in leisure time walking minutes ($p< .05$) and negatively related to weight change ($p < .01$) 6. Number of accelerometer uploads were not related to PA or weight
4. Task enjoyment<br>5. Motivation	1. Time in plank: greater in I3 64.48 (±20.19) seconds, p=0.1, compared to I1 54.98 (±17.12), I2 54.36 (±16.50) seconds, and C 50.56 (±18.43) seconds;<br>males had greater times spent in plank over all conditions, p=.006	2. Mean scores were overall higher in the group dynamics-based conditions than the standard conditions ($p< 0.05$);<br>significant differences between conditions in all dimensions of cohesion ($p<.001$ to $p=.04$), except for attraction to group-task ($p=.06$) 3. No significant differences	4. No significant differences<br>5. No significant differences

4. Feasibility and acceptability (acceptability of contents of materials and technologies used for the intervention)<br>5.Fidelity (receipt of intervention materials and interaction with it)	1. Sedentary behavior: decrease for I1 (71 +379 min/day) compared to I2 (increase of 118 +753 min/week), p=0.026;<br>light PA: greater increase for I1 (95 +210 min/week) compared to I2 (59 +401 min/week), p=0.024 ;<br>moderate-lifestyle PA: increase for I1 (27 +112 min/week) compared to I2 (decrease of 35 +248 min/week), p<0.001;<br>self-reported PA: increase for I1 (62 +169 min/week) compared to I2 (7 +194 min/week), p=0.015;<br>steps/day: No significant differences, but decrease for I1, p=0.016;	2. Self-regulation for PA higher increase in I1 (1.51 +0.63) compared to I2 (0.96 +0.69), p=0.001;<br>social support from family for PA higher increase for I1 (3.36 +9.44) compared to I2 (2.71 +10.23), p= 0.44;<br>outcome expectations for PA higher increase for I2 (0.26 +0.49) compared to I1 (0.15 +0.32), p=0.001<br>3. No significant changes	4. Feedback from I1 was overall more favourable than from I2, I1 reported more often than I2 that they gained helpful knowledge from the intervention and were motivated to increase PA; 100% of I1 would recommend the program and 87% of I2<br>5. 100% viewed weekly PA promotion posts and group discussion topics during the first half of the study, but only 64-86% during the second half, half of I1 participants viewed all PA promotion materials and median number of participant comments

6. Feasibility (recall, use, and satisfaction with the program) 7. Engagement (logins, step logging frequency, and team member interactions)	1. Accelerometer-derived MVPA: no significant differences, but within group increases maintained to follow-up, I1 from 147-173 min/week, I2 from 195 - 227 min/week, and C from 137 - 160 min/week, p<0.01;<br>self-reported walking: no significant differences	2. No significant differences<br>No significant differences<br>significant differences<br>No significant differences	3.<br>4. No<br>5.	6. App helped increasing PA (65%) and was rated to be of interest for postpartum women (62%)<br>7. App was visited on average 26 times/50 days and steps were logged for 48/50 days, mean number of virtual gifts sent to teammates was 7, and the mean number of posts on the group message wall was 9
2. User retention and engagement with the app (steps logged, daily logins, number of interactions with team mates)<br>3. Usability app (participants' opinions regarding the app including: ease of use, appeal to the target audience, interest and impact of the key features, and suggestions for improvement)<br>4. Team forming process (number of invitations sent/declined, number of days needed to reach minimal team members/to finalise team)	1. Total PA increased from 294 (±307) min/week to 471 (±437) min/week, p=0.01			2. Retention: 86%;<br>engagement: mean number of weekly step entries ranging from 6.9 (± 0.3) to 5.5 (± 2.8) was high;<br>most used feature, out of 25, 18 participants logged steps for all 28 days, 22 participants logged steps for more than 21 days, 24 logged steps for more than 14 days; overall engagement: decreased towards the end of the intervention and log-in rates dropped<br>3. Ease of use & navigation: the team feature and montoring of progresses were strengths of the app; main issues lied within the design not fitting adequatly to every device;<br>minor issues with the registration process could be resolved during the recruitment process<br>4. Teams took a median of 13 days to form, participation rate was 68.4 %

8. Feasibility (log-ins to the service, use of the different service sections, feedback of participants)	1. Significant differences were only observed for the last weeks of the trial seperately, p< 0.05 to p< 0.001	2. No significant differences<br>No significant differences<br>significant differences<br>significant differences<br>significant differences<br>No significant differences	3.<br>4. No<br>5. No<br>6. No<br>7.<br>8. Functionalities related to PA were rated most important (by 90%), other mentioned features were instructions, test, and goals on PA, as well as general information on health (11%); compliance was limited, with 64.4% participants visiting the service, only 47.2% logged in more than once, and only 16.4% more than 5 times
a)<br>5. Process evaluation (compliance, engagement, feedback)<br>b)<br>6. Engement with Facebook posts (likes, comments, poll votes, photo uploads)	a)<br>1. MVPA: larger increase in I (140 ±50 min/week) compared to C (91 ±47 min/week), p=0.04;<br>b)<br>2. Compliance with the running program: positively associated with the total engagement with the Facebook intervention, p= 0.04	a)<br>3. No significant differences<br>No significant group differences; improvements for social support in both groups compared to baseline, p=0.02	4.<br>a)<br>5. Completed running sessions: 72% ±7.2 for I compared to 60% ±8.1 for C; feedback: generally positive, with 63% agreeing that the Facebook group helped them complete the running program;<br>75% rated the posts supportive, 66% relevant and motivating; participant-initiated posts mostly rated supportive (69%), relevant (59%), and motivating (59%)<br>b)<br>6. Posts received a mean of 6.6 interactions, most commonly in the form of likes, and least commonly in the form of photo uploads; moderator-initiated polls prompted highest levels of engagement, p = 0.0005 to p = 0.006; moderator-initiated post generated more engagement than participant-initiated posts, p ≤ .01

3. Engagement/use (number of visits to the app, participants' step-logging patterns, number of virtual gifts sent, and number of posts on the message walls)<br>4. Feasibility (/ceptions of the app/of specific features, /ceptions of the impact of the program)	1. Overall MVPA: larger increase for I (248 ±59 min/week) compared to C 113 ±43 min/week), p=0.03 but no significant differences at follow-up; "high intervention dose" participants (>18 log-ins) had higher increase than "low dose" participants, p=0.04;<br>walking time: larger increase for I (205 ±38 min/week) compared to C (50 ±23 min/week), p<0.001 but no significant differences at follow-up;<br>no significant differences for any other types of PA outcomes	2. No significant differences	3. 94% used the app at least once, 55% logged steps for all 50 days of the program as intended, 69% logged steps on 36 days or more; 31% met or exceeded the intervention target of 500,000 steps in 50 days;<br>participants sent a mean of 4.8 (± 6.3) gifts, and made a mean of 2.7 (± 3.4) wall posts to their team discussion wall 4.<br>Generally positive feedback about the app, a majority found it to be user friendly and easy to navigate and liked the overall presentation of the app, page to log steps was found to be useful, half of respondents reported influence of teammates to improve their exercise regimen and provision of social support through the app
4. Feasibility (recruitment rate, Fitbit Flex weartime, number of participants completing online questionnaires > 90%)<br>5. Facebook engagement	1. No significant differences	2. Social functioning improved for C (84.7) compared to I (83.9), p=0.04<br>3. Introjected motivation improved for I (1.7) compared to C (1.4), p<0.05	4. Fitbit wear time: 71.5% ;<br>90% completed study<br>5. No relation between engagement and PA;<br>89.7% joined the FB group;<br>92.3% saw at least one group wall post;<br>65.4% commented at least once;<br>50% liked at least one post
	1. MET (min/week) higher in I (3762.84 ±2988.60) compared to C (2146.84 ±1586.23), p=.010; walking minutes/week higher in I (102.19 ±67.62) compared to C (69.69 ±45.75), p=.028	2. Total PMS score decreased in I (240.94 ±74.52) compared to C (296.28 ±71.00), p=.003;<br>PMS symptoms out of 18, 13 improved significantly in favour for I compared to C;only 1 symptom change showed favourable results for C compared to I; no significant differences in the 4 remaining symptoms	

	1. Number of daily steps was significantly higher in I than in C throughout the six weeks of intervention (11.563 ±709 vs. 9006 ±689 steps/ day, p<0.05);<br>amount of PA (daily total METs/h) at an intensity of ≥3 METs was also significantly higher in I than in C	2. Body fat and WC were significantly reduced only in I;<br>changes in the amount of PA and in body fat were significantly correlated	
10. Feasibility and acceptability (recruitment and enrollment rates, loss to follow-up)	1. Daily steps: greater change for I (+3589.14) compared to C (+480.27), p<0.001	2. I lost an average of 3.58 kg (3.04%) more than C, p<0.001<br>3. Greater reduction for I (-1.39) compared to C (-0.26), p<0.001, 4. Reduction for I (-3.96) compared to C (-1.12), p=0.01<br>5. Greater reduction for I (-10.0) compared to C (-3.97), p<0.05 6. No significant differences 7. Healthful eating: improvement for I (-3.12) compared to C (-0,92), p<0.001;<br>fatty food sore: reduction for I (-3.05) compared to C (0.49), p=0.01<br>8. No significant differences<br>9. Greater change for I (12.23) compared to C (5.18), p=0.006	10. High rates of retention at minimally supported phase in I
11. Use and acceptability<br>12. Retention and feasibility (frequency of MapMyFitness use, positive/negative features, enjoyment and technical problems associated with the app, opinions on the Facebook-delivered health education intervention)	1. MVPA: no changes for I, decrease for C<br>sedentary behavior: decrease for I, increase for C	2. No significant changes<br>3. Decrease of 1.2 kg for I and 0.6 kg for C<br>4. Decrease of 0.8% for both groups<br>5. No notable changes<br>6. No notable changes<br>7. Increase of 21.0% for I and 15.4% for C<br>8. No notable changes<br>9. Decrease of 4.8% for both groups<br>10. No notable changes	11. Implemented the tips 1-3 times/week; app use 1.71 times/ week;<br>future usage 100% would recommend future implementation<br>12. Retention: 95%;<br>Facebook-delivered health education tips: mostly rated “somewhat helpful”;<br>ease of use and workout feedback: was rated positively

13. Use and acceptability ("liking" and seeing posts on Facebook, frequency of Facebook tip implementation)<br>14. Retention and feasibility (helpfulness; usefulness and ease-of-use of smartwatch and positive/negative features)	1. MVPA: I increased by 110.7% (+194.8%), C by 44.6% (+124.5%);<br>no other notable changes	2. No significant changes<br>3. Decrease of 0.6 kg in I and 0.5 kg in C<br>4. Increase of 2.2% in I and 0.3% in C<br>5. Improvement in I (3.3 beat/minute decrease) but not in C (1.8 beat/minute increase) 6. Increase of 33.7% for I and 28.7% for C<br>7.Increase of 36.5% for I and 19.0% for C<br>8. No notable changes<br>9. No notable changes<br>10. No notable changes<br>11. Increase of 13.7% for I and 12.3% for C<br>12. Decrease for I (41.0 calories, -0.9%) and for C (143.3 calories, -4.6%)	13. Adherence: 89.8% (+ 21.8%) in I, 84.4 (+ 22.3%) in C, implemented the tips 1-3 times/week<br>14. Retention: 84.2% in I, 100% in C ;<br>Facebook-delivered health education tips: mostly rated "helpful"; Smartwatch: "somewhat helpful" to "helpful" in assisting to increase PA;<br>ease-of-use: rated as "somewhat easy";<br>future usage: 100% would recommend future implementation
12. Use and acceptability (helpfulness of FB tips, frequency/duration of MapMyFitness use)<br>13 usability (positive/negative features or technical problems with the app, and application enjoyment)	1. MVPA : increase (2.6 min);<br>Light PA and sedentary behaviour: decrease<br>daily step counts: increased by 1,657 steps,<br>daily activity-related energy expenditure: increased by 87 kcal	2. No notable changes<br>3. Decrease of 2.4 kg<br>4. No notable changes<br>5. No notable changes 6. Increase of 3%<br>7. Increase of 0.56 points<br>8. Increase of 0.15 points<br>9. No notable changes<br>10. No notable changes<br>11. Limitations in engaging in social roles decrease of 0.62 points;<br>pain intensity decrease of 0.29 points;<br>physical functioning decrease of 0.16 points;<br>anxiety decrease of 0.25 points; ;<br>depression decrease of 0.17 points,<br>sleep disturbances decrease of 0.19 points	12. App usage: 3.75 times/week, for 39.7 min at midpoint and 35 min at postintervention; Facebook group usage: 16 posts were made by participants (content was mostly related to participant's PA); 7.4 (± 0.9) of 10 read each post;<br>future usage: 100% would recommend future implementation<br>13. Overall satisfaction with the app: small technical difficulties, lesser satisfaction with specific app features, health education tips rated useful by most participants (except for one)

12. Use and acceptability (frequency of Facebook tip implementation, weekly frequency/mean duration of smartwatch use during exercise) 13. Usability (helpfullness, enjoyment and negative features)	1. No significant group differences; slight improvements for both groups compared to baseline for all PA outcomes, except sedentary behavior	2. No significant differences 3. No significant differences 4. No significant differences 5. No significant group differences, but both groups demonstrated reduced heart rate compared to baseline<br>6. No significant group differences<br>7. Social support lower in I (2.7 ±1.3) compared to C (3.0 ±1.1), p=0.05<br>8.No significant differences<br>9.Higher in I (2.0 ±0.5) compared to C (1.8 ±0.4), p=0.04<br>10. No significant differences<br>11. No significant group differences, but I decreased social role limitations and sleep disturbances, and increased sleep quality compared to baseline	12. Facebook health tips were implemented about 1.2 (±1.0) times/weekly; Smartwatch usage: average wear time of 6–7 days/week; weekly mean frequency and duration/session for smartwatch use was 4.55 sessions/week and 53.9 min/session<br>13. Facebook health tips were mostly enjoyed;<br>just 7 of 12 participants reported enjoying the smartwatch, due to negative features of the device (too big)
5. Intervention adherence (number of times participants provided their steps data)<br>6. Engagement in the Facebook group (posts, type of posts)	1. steps higher in I (12.472 ±2816.61) compared to C (10,135.64 ±3316.37) , p<0.001	2. No significant differences<br>3. No significant differences<br>4. No significant group differences, improvement for I comapared to baseline ([illegible]1.1 cm), p=0.003	5. Did not predict increase in steps/day<br>6. Amount of posts: decreased over time (from <50 in week 1 to <20 in week 8); did not predict increase in steps/day

5. Process evaluation (walking adherence, social networking participation, intervention satisfaction, treatment contamination)	1. No significant group differences; improvements for the full sample for MVPA (21.0 mins/week, 95% CI)	2. No significant group differences, full sample improved at follow-up<br>3. No significant group differences, full sample improved at follow-up<br>4. No significant group difference, full sample improved	5. Walking adherence: 71.6% (± 32.1%) completed walking sessions; return of 9.1 (± 3.7) of 12 possible weekly walking logs;<br>social network participation: participation in 3.9 (± 2.6) of the 12 social networking activities, only 8.7% of participants posted a discussion comment on the networking site;<br>intervention satisfaction: 91.8% would recommend programm to others
	1.Sedentary time/very light PA (% of time on weekdays): lower amounts for I2 (56%) compared to C (65%), p=0.021;<br>no significant differences for any other PA outcomes	2. No significant differences	

5. Feasibility (Intervention receipt, use of intervention components, intervention satisfaction, adverse events, contamination, sustainability)	1. No significant group differences, but a significant improvement over time, p=0.04	2. Higher improvement for I compared to C (F= 3.45, p=0.04)<br>3. No significant differences<br>4. No significnt differences	5. Intervention receipt: 12 participants never signed up for Meetup, logins averaged 58.38/week (±11.62);<br>use of intervention components: 55.0% did not attend any community events and 42.9% never commented on the Meetup website;<br>participants attended 2.07 (±3.11) dog walks; 28.6% reported they read all newsletters; 39.3% of participants reported using the activity monitor 5 times or less; 35.7% reported daily or almost daily use; 60.7% of participants never recorded their steps on the website; intervention satisfaction: rated lowest for schedule of neighbourhood walks (2.97 ±1.27) and highest for the activity monitor (4.00 ±1.26);<br>46.4% reported that none of the dog walks were scheduled at a convenient time;<br>adverse events: 6 (reported in the AHA condition) were possibly related to dog walking, as well as 5 dog-related adverse events (2 in the Meetup condition);<br>contamination: no AHA participants reported to know anyone from the Meetup group or had any contact with their intervention; sustainability: Meetup groups were self-organized after the intervention and opened up, membership grew and regular dog walks continued
4. Study participation<br>5. Acceptability	1. Total increase of 3218 steps, 107 steps/day, p=0.005	2. Decrease in teen- and parent-reported symptoms, ranging from -0.4 to -0.8 on the VADPR-scale, p< 0.0001<br>3. No significant changes	

	1.Students in I had 3.51 times higher likelihood to be sufficiently active after one month compared to C (92% vs.82.8%), p=0.024;<br>students active at baseline had 5.44 times higher likelihood to be sufficiently active after one month compared to those who were inactive (93.4% vs.74.1%), p<0.001		
2. Engagement (log entries, frequency/length of app usage, retention)<br>3. Usability	1. Overall no significant changes, subgroup analysis of lower PA group shows increase of 3025 steps compared to baseline, p=0.008		2. Retention: 82%;<br>Usage of Fitbit: was higher than app and social features; App usage: decreased over time; 4 participants did not use the app at all throughout the study; 3. Mean system usability score was 60.1 (+ 19.2), which indicates a low usability
10. Acceptability (number, date, type, and views of posts on Facebook, number of participants receiving pedometer and app instructions and using them, number of participants not willing to use any intervention tool) 11. Feedback (components participants liked most, those that were less useful, suggestions for future interventions)	1. Daily steps: decrease from 8496 (±2,528) to 8136 (±2,395), p=0.04;<br>MVPA (median): decrease from 19 to 13 (p=0.01);<br>no significant changes for any other PA outcomes	2. Fruit and vegetables (% of total daily energy intake): increase from 16% (±8%) to 20% (±8%), p=.04, no maintenance at follow-up 3. No significant changes 4. No significant changes<br>5. No significant changes<br>6. No significant changes<br>7. No significant changes<br>8. No significant changes<br>9. No significant changes	10. Usage: 60% of participants used at least 1 tool;<br>68.4% used the app less than once a month or never, and they used the pedometer at least once a week (57.9%);<br>47.4% engaged with the Facebook group at least once a week; 57,9% reported that they set diet-related goals at least once a week, PA goals were set less frequently;<br>11. Evaluation: pedometers and Facebook content were considered good motivations;<br>app was not reported to be useful;<br>intervention raised awareness for current habits;<br>focus was on change of dietary patterns as changing two behaviors at once was considered to be difficult

a)<br>10. Adherence and acceptability (intervention exposure, attention, recall, satisfaction with intervention components and recommendation of intervention to peers, goal-setting frequency, number of PA entries, number of walking steps entries)<br>c)<br>11. Facebook engagement (interaction within Facebook groups and content)	a)<br>1. MVPA: no significant group differences; vigorous PA: participants who scored high for goal-setting had an higher increase in vigorous PA (82.7 ±16.9 min/week vs. 30.7 ±17.7 min/week), p=0.043;<br>light PA: larger increase in I (163.6 min/week) compared to C (28.5 minutes/week, p=0.032<br>b)<br>2. MVPA: across groups, changes were predicted by changes in total social support (β = 6.21 ±1.65), p=0.0003 and social support from friends (β = 10.35 ± 3.68), p=0.006;<br>self-monitoring was positively related to change in MVPA (β = 57.22 ±19.47), p=0.004<br>c)<br>3. MVPA: greater change for Facebook "Active group" (18.5 min/week) compared to the "Somewhat Active" group (-48.4 min/week), p=0.009 and the "Inactive group" (-46.5 min/week), p=0.042;<br>light PA: making a post on Facebook was associated with an increase of approximately 12 min/week in light-intensity PA (β = 11.77, p=0.049)	a)<br>4. No significant differences<br>5. No significant differences<br>6. No significant group differences, but I reduced weight compared to baseline (-2.1 kg), p=0.004<br>b)<br>7. Greater decrease for I (- 0.38) compared to C (-0.01), p=0.025<br>8. Higher decrease for I (- 0.47) comparted to C (1.46), p=0.039<br>9. No significant differences	a)<br>10. Both groups agreed that posts were easy to access and effective in gaining information about PA;<br>half (46.9%) of I participants would recommend the intervention, compared to 61.8% of C participants;<br>no significant differences for adherence, 62.5% of I participants and 79.4% of C participants reported usually reading some to all/most of the Facebook messages;<br>both groups reported using various Facebook group features 1 to 2 days a week (visiting the Facebook group, seeing a FITNET group post in their news feed, read FITNET group discussions)<br>c)<br>11. Facebook engagement: no group differences, participants that made any posts decreased over time in both groups;<br>C participants were more likely than I participants to agree that group discussions caused them to become physically active, p = 0.040, and that group members were supportive, p = 0.028; participant-initiated posts elicited significantly more response comments (p = 0.003) and likes (p = 0.002)

4. Vlog exposure, liking of the vlogs 5. /ceived closeness with vloggers	1.Total PA: greater improvement for C than for I1, p=0.002	2. No significant changes<br>3. Descriptive norm for PA: higher in I1 compared to I2, p=0.009<br>4. No significant differences<br>5. No significant differences<br>6. No significant differences	4. Exposure and liking: participants in I1 watched (15.69 ± 20.60 times) and liked (69.09 ± 30.42) the vlogs more often than participants in I2 (7.21 ± 14.60; 40.20 ± 32.72), p<0.001<br>5. Perceived closeness: higher for I1 (4.68 ± 1.61) compared to I2 (3.46 ± 1.97), p< 0.001;<br>intervention messages created by acquainted peers increase engagement with the materials;
	1. PA: higher in I1 (3.47 ±0.8) and I2 (3.43 ±1.05) compared to I3 (2.0 ±0.87) and C (1.86 ±1.07), p<0.1 for all;<br>no significant difference in increase between I1 and I2	2. Competence: higher in I1 (5.09 ± 1.03) and I2 (5.13 ± 0.82) compared to C (3.61 ± 1.41), p< 0.05;<br>enjoyment: higher in I3 (5.65 ± 0.81, p< 0.01) compared to I1 (5.2 ± 0.73), I2 (5.17 ± 0.92) and C (4.53 ± 1.2); vitality: increase for all four groups, p< 0.01<br>3. No significant differences	

2. Use/social encouragement	1. Steps/day: I walked an average of 15.085 (±8209.65) steps/day;<br>short-term effect of social encouragement on steps/day: all three forms had significant positive effects on the next day's exercise level (p<0.05) in descending order thumbs-up, comments, posts	2. Number of posts by friends/day= 0.3467 (±3.68); number of comments/day= 0.00973 (±0.27); number of thumbs-up/day= 0.00964 (±0.33)
2. Facebook group usage<br>3. Program evaluation and feeback (overall ex/ience/specific components)	1. No significant group differences for any PA outcome	2. Usage: generally low, no significant differences;<br>rate of post views high with 80 % seen by the total sample<br>3. Feedback: 70% were satisfied with the overall experience;<br>55% found the content to be interesting or useful (45%);<br>55% visited the group once or twice in a week ;<br>45% interacted with the posts;<br>50% learned about PA ;<br>70% reported an influence on their PA

2. Engagement (average XP points gained / day)	1. Steps/day: relative increase of 34.8% (from 5678 ±2833 to 7654 ±3616 steps/day) $p<0.001$; from all subgroups, participants 30 years or older had the highest increase (3320 steps/day), $p<0.05$;<br>% of days with >10 000 steps / day: increased from 15.3% before to 27.5% after, $p<0.001$;<br>dose-response: on average, every 10.000 XP points gained in Pokémon GO were associated with 2134 additional steps / day, $p<0.001$	2. Mean Pokémon GO XP points/day: 10.293 (± 8651) points suggesting study participants captured as many as 100 Pokémon/day
	1. Attended exercise classes higher in comparison conditions I2 & I3 (5.1 and 5.5 times) compared to I1 and C (2.4 to 2.9), $p<0.001$;<br>all observed effects can be attributed to the social comparison component, increased activity levels by 62%, $p=0.03$	

1. Class attendance: no significant differences between I1 and C or I1 and I2; longer sustainment of class enrollment in I2 compared to I1 and C, $p<0.001$;
likelihood of enrollment:
- in 4 or more classes: 60% higher in I2 compared to C (OR= 2.4, $P<0.01$)
- in 5 or more classes: 80% higher in I2 compared to C (OR= 2.7, $P<0.001$)
- in 6 or more classes: 170% higher in I2 compared to C (OR= 4.1, $P<0.001$);

exercises: increase for I2 (1.6 $\pm$0.3 days) compared to C (0.08 $\pm$0.3 days), $p=0.02$

s. Comments

No significance tested because of sample size

No significance tested, only descriptive results

No significance tested, only descriptive results

No significance tested, only descriptive results

MVPA: N= 300
Other health outcomes: N= 308
